# The impacts of vitamin D supplementation in adults with metabolic syndrome: A systematic review and meta-analysis of randomized controlled trials

**DOI:** 10.3389/fphar.2022.1033026

**Published:** 2022-10-05

**Authors:** Kai-Jie Qi, Zhong-Tao Zhao, Wen Zhang, Fang Yang

**Affiliations:** School of Laboratory Medicine, Hubei University of Chinese Medicine, Wuhan, China

**Keywords:** vitamin D, metabolic syndrome, randomized controlled trials, meta-analysis, inconclusive results

## Abstract

**Background:** Studies have shown the association of vitamin D status with the development of metabolic syndrome (MetS), which has attracted an extensive research interest with inconsistent results. Therefore, we hypothesized that vitamin D supplementation (VDS) will benefit adults with MetS.

**Aims:** To test our hypothesis, we performed a meta-analysis to evaluate the effect of VDS on MetS in adults using relevant biomarkers such as anthropometric parameters, blood pressure, blood lipid profile, glycemia, oxidative stress and vitamin D toxicity (VDT).

**Methods:** Randomized controlled trials published in PubMed, Web of Science, embase and the Cochrane Library between 2012 and 2022 on the effect of VDS on MetS in adults were searched. The language was limited to English. A meta-analysis performed using RevMan 5.4 and Stata 14.0 software, sensitivity analysis, and evaluation of the risk of bias and general quality of the resulting evidence were conducted.

**Results:** Eventually, 13 articles were included in this meta-analysis. Overall, VDS significantly increased the endline serum 25-hydroxyvitamin D levels as compared to the control [MD:17.41, 95% CI (14.09, 20.73), *p* < 0.00001]. VDS did not affect waist circumference, body mass index, body fat percentage and VDT biomarkers, but decreased waist-to-hip ratio and blood pressure (*p* < 0.01). VDS significantly decreased fasting plasma glucose (FPG) [MD: 3.78; 95% CI (−6.52, −1.03), *p* = 0.007], but did not affect the levels of blood high-density lipoprotein cholesterol (HDL-C), low-density lipoprotein cholesterol (LDL-C), total cholesterol (TC), and triglyceride (TG). Pooled estimate of nine papers indicated a significant reduction of fasting insulin (FI) (*p* = 0.006), and homeostasis model assessment of insulin resistance (*p* = 0.0001). The quantitative insulin check index levels were moderately increased (*p* = 0.007) without any impact on the glycosylated hemoglobin type A1C (HbA1c). For the oxidative stress parameters, VDS significantly lowered the levels of malondialdehyde and hypersensitive C-reactive protein (*p* < 0.05).

**Conclusion:** Results of this meta-analysis demonstrate that VDS only reduces insulin resistance and hypertension but not the blood lipid profile and HbA1c. It appears that the evidence for the benefit of VDS in adults with MetS is inconclusive. Further clinical studies are still needed.

## 1 Introduction

As people’s living standards have generally increased, metabolic syndrome (MetS) has progressively emerged as a major public health issue that wreaks havoc on people’s health (Saklayen, 2018). MetS is a metabolic disorder associated with abdominal obesity, insulin resistance, hypertension, and dyslipidemia that increases the risk of developing type 2 diabetes mellitus (T2DM) and cardiovascular diseases (CVDs) (Melguizo-Rodríguez et al., 2021). The diagnostic criteria for MetS were established mainly by the World Health Organization (WHO) (Alberti and Zimmet, 1998), National Cholesterol Education Program’s Adult Treatment Panel III (NCEP-ATP III) (Grundy et al., 2005) and International Diabetes Federation (IDF) (Zimmet et al., 2005), which are used for surveys and health care plans. The diagnosis of MetS has six indices including waist circumference (WC), fasting plasma glucose (FPG), triglyceride (TG) levels, high-density lipoprotein cholesterol (HDL-C) levels, total cholesterol (TC) levels, and blood pressure (BP) (Fahed et al., 2022). In recent years, the prevalence of MetS is increasing dramatically across the world (O'Neill and O'Driscoll, 2015; Faraji and Alizadeh, 2020; Melguizo-Rodríguez et al., 2021). MetS is estimated to be present in 25% of the world’s population, with substantial variation based on gender, age, and race (Melguizo-Rodríguez et al., 2021). As CVDs are the most common cause of mortality and morbidity globally, it is crucial to investigate how MetS contributes to it (Fahed et al., 2022). Positive family history, genetic predisposition, aging, obesity, stress, low cardiorespiratory fitness, low birth weight, western diet, drinking sweetened beverages, alcohol consumption, cigarette smoking, physical inactivity, low socioeconomic status and growing urbanization are some of the interrelated risk factors for MetS (McCracken et al., 2018; Bovolini et al., 2021). Additionally, some medications, such as antipsychotics, sedative-hypnotics, and antidepressants, may contribute to the development of MetS (Mitchell et al., 2013; Gramaglia et al., 2018). Among so many influencing factors, dietary factor is one of the most important ones affecting the rates of MetS, such as high-calorie and high-fat diets (Castro-Barquero et al., 2020).

As a fat-soluble prohormone, vitamin D is associated with skeletal growth and bone health and may potentially play crucial roles in the immunological and other systems. Cholecalciferol, often known as vitamin D3, is the primary source of vitamin D in the body. It is generated from 7-dehydrocholesterol under the action of ultraviolet rays on the skin, and obtained from animal diet (Raposo et al., 2017). Ergocalciferol or vitamin D2 can be obtained from vegetable foods. These two sources of vitamin D precursors are sent to the liver to synthesize 25-hydroxycholecalciferol or calcidiol, and ultimately the active form of 1, 25-dihydroxycholecalciferol or calcitriol in the kidney (Melguizo-Rodríguez et al., 2021; Theik et al., 2021). In recent years, the incidence of vitamin D deficiency has been on the rise, which has become a concern of global health (Mukhopadhyay et al., 2019). Vitamin D deficiency due to insufficient dietary sources and lack of vitamin D-fortified foods leads to rickets in children and osteomalacia in adults. Vitamin D insufficiency (25(OH)D concentration at 21–29 ng/ml and deficiency (25(OH)D concentration at less than 20 ng/ml are concerns of public health (Pfotenhauer and Shubrook, 2017). Around the world, the average per capita vitamin D supply is estimated to be < 60, 64-120, 124-220, 224-300, 304-400, and >400 IU/d in 40, 60, 70, 4, 2, and 2 nations, respectively (Cashman, 2021). The vitamin D intake recommendations for an adult are ranging from 400 to 1000 IU/d (10–25 g/d) according to different organizations (Holick et al., 2011; Ross et al., 2011; Efsa Panel on Dietetic Products et al., 2017bib_EPDP_2017). However, a greater risk of exogenous hypervitaminosis D, with symptoms of hypercalciuria and hypercalcemia and also known as vitamin D toxicity (VDT), may arise from increases in vitamin D supplementation (VDS) in the general population and therapeutic prescriptions lacking medical supervision (Dudenkov et al., 2015). VDS has hormonal, anti-inflammatory, anti-apoptotic and anti-fibrotic activities, with preventive action against CVDs, T2DM, tumors, Alzheimer’s disease and MetS (Melguizo-Rodríguez et al., 2021). The association between VDS and MetS is controversial. The benefits of VDS in the treatments of MetS and its disorders connected include improved arterial stiffness, mitochondrial oxidation and phospholipid metabolism; increased lipoprotein lipase activity, peripheral insulin sensitivity and *β*-cell function; decreased inflammatory cytokines and parathyroid hormone levels, and renin-angiotensin-aldosterone system activity (Mahmood et al., 2016; Teixeira et al., 2018; Chen et al., 2019; Ferreira et al., 2020; Xu et al., 2020). Zhu and Heil reported that serum 25-hydroxyvitamin D (25(OH)D) level was linked to the risk factors for MetS, and that 1 ng/ml rise in serum 25(OH)D level was related with a considerable decrease in TC and LDL, as well as a 54% reduction in the risk of MetS (Zhu and Heil, 2018). However, some studies reported no significant association between VDS and MetS in adults (Piantanida et al., 2017; Mansouri et al., 2018; AlAnouti et al., 2020; Ganji et al., 2020; Wang et al., 2020; Zheng et al., 2020). Similarly, Mehri et al. claimed that a definite causative relationship between low serum 25(OH)D levels and MetS could not be demonstrated clearly due to the lack of long follow-up studies (Mehri et al., 2019).

Therefore, the seemly link between VDS and MetS still needs evidence to show whether VDS is beneficial in treating MetS. A large and growing body of literature has demonstrated the beneficial effects of VDS on body components in patients with MetS. A meta-analysis done by Ostadmohammadi et al. demonstrated the beneficial effects of VDS on improving glycemic control, HDL, and hypersensitive C-reactive protein (hs-CRP) levels in patients with CVDs (Ostadmohammadi et al., 2019). A cross-sectional study performed by Mutt et al. showed that the vitamin D-supplemented participants had a considerably reduced incidence of MetS and its components in comparison to the non-supplemented participants (Mutt et al., 2019). Meanwhile, Vimaleswaran et al. discovered that an increase in plasma 25(OH)D concentrations may decrease the prevalence of hypertension (Vimaleswaran et al., 2014). Another meta-analysis conducted by Golzarand et al. revealed that the daily VDS for more than 800 IU doses significantly reduced both systolic blood pressure (SBP) and diastolic blood pressure (DBP) in subjects older than 50 years. Moreover, hypotensive effects were observed in both healthy and hypertensive subjects (Golzarand et al., 2016). However, in contrast to the above points, Makariou et al. commented that there is no relationship between serum 25(OH)D levels and MetS parameters, such as body mass index (BMI), blood pressure, lipids, glucose, insulin and homeostasis model assessment of insulin resistance (HOMA-IR) levels in adolescents with obesity compared with normal-weight controls (Makariou et al., 2020). Similarly, a randomized controlled trial (RCT) undertaken by Tamadon et al. discovered that no significant effect of VDS on lipid profiles and other biomarkers of inflammation and oxidative stress in diabetic hemodialysis patients compared with the placebo (Tamadon et al., 2018). So far, there is still debate regarding whether VDS and MetS are related due to the lack of conclusive scientific evidence. In order to determine the link between VDS and MetS, we thus try to statistically analyze these RCT data via a meta-analysis. This meta-analysis and systematic review were aimed to reach a definite conclusion about the effect of VDS on components of MetS.

## 2 Methods and materials

### 2.1 Review design

This systematic review was registered in PROSPERO (CRD42022340128) and conducted according to the Preferred Reporting Items for Systematic Reviews and Meta-Analyses (PRISMA) statement (Moher et al., 2010). Ethical approval was not required for the current study.

### 2.2 Search strategy

The search strategy considered three key concepts: vitamin D, MetS and adults. Medical Subject Headings (MeSH) and keywords were mapped for each concept. Then we searched in PubMed in order to get more comprehensive search terms. Vitamin D, cholecalciferol or vitamin D3, ergocalciferol or vitamin D2, calcidiol and calcitriol were among the search phrases, along with MetS in adults. A computer search of all published RCTs about the effects of VDS on adult with MetS in PubMed, Web of Science, embase, and Cochrane Library from 2012 up to 2022. Language was restricted to English. Supplementary Material S1 presents the specific search procedures with each database and the associated search results. We also manually combed through the reference lists of all eligible articles and prior reviews on pertinent topics to identify additional studies. For unpublished studies, the Google search engine recommended by the current Cochrane Collaboration guidelines and the clinical trial registry database were used. Two investigators (KJQ, WZ) independently searched papers, screened titles and/or abstracts of the retrieved studies. Then, through reviewed the full texts of potentially eligible studies to select articles that meet the inclusion and exclusion criteria. A calibration exercise was first carried out to make sure the validity of the study selection process. Consensus was reached to resolve disagreements or with assistance from a third reviewer (FY).

### 2.3 Inclusion and exclusion criteria

This systematic review included RCTs that conducted on adults (aged >18 years) with MetS. In the RCTs, the intervention group was supplemented with any form of vitamin D3 or D2. The following inclusion criteria were used: 1) RCTs that compared VDS with a placebo, and, RCTs involving a co-intervention were included if both studies received the same co-intervention. 2) Human study subjects. 3) Articles published in English language. 4) RCTs investigated at least one component of the MetS (anthropometric parameters, blood pressure measurement, blood lipid and glycemia profile). 5) Only RCTs lasting at least 4 weeks were included to ensure that the interventions had enough time to have an effect. 6) The data reported in the literature should be continuous measures, as the mean ± standard deviation (SD) of the change value. Studies were excluded if their subjects are minors, healthy people, or some underlying diseases that can affect vitamin D metabolism, such as end-stage renal disease, and hyperparathyroidism. In addition, studies with an intervention duration shorter than 4 weeks or longer than 28 weeks should also be excluded, as intervention duration is decisive. Finally, studies were excluded if they were publications from letters, meta-analysis or reviews. If there were duplicate studies, we made sure the most current or comprehensive one was included.

### 2.4 Data extraction

The extracted data included basic characteristics (including the first author’s name, year of publication, county, study design, representativeness of the study population and funding sources), gender, age and health status of subjects, sample size, criteria for MetS, intervention (the unit and dose of VDS, duration time) and control, outcome measures, as well as records used to evaluate the risk of bias. The outcome measures included: 1) anthropometric parameters and blood pressure including BMI, WC, waist to hip ratio (WHR), body fat percentage (BF%), SBP, DBP, 2) serum 25(OH)D level, 3) blood lipid profile including HDL-C, low-density lipoprotein cholesterol (LDL-C), TC and TG, 4) blood glycemia aspect including FPG, fasting insulin (FI), glycated hemoglobin (HbA1c), HOMA-IR and quantitative insulin check index (QUICKI), 5) oxidative stress parameters including malondialdehyde (MDA) and hs-CRP, 6) VDT biomarkers including parathyroid hormone (PTH) and serum calcium. Three investigators (KJQ, ZTZ and WZ) independently and in duplicate retrieved data and have assessed all qualifying RCTs by using standard forms and the Cochrane Collaboration risk of bias tool (Mansournia et al., 2017). A calibration operation was first performed to validate the data extraction procedure. Data were cross-checked and any dispute was settled via discussion with the corresponding author (FY). To see whether any data were missing or inadequate, we attempted to email the authors of the listed papers.

### 2.5 Quality assessment of studies

The risk of bias in the included RCTs was assessed according to the Cochrane criteria (Cumpston et al., 2019). The contents of risk assessment include random sequence generation, allocation concealment, blinding of participants and personnel, blinding of participants and outcome assessors, incomplete outcome data and selective outcome reporting. For other bias, funding was evaluated. Two researchers (KJQ and ZTZ) independently and consistently assessed the risk of bias in the included RCTs following the Cochrane criteria. Disagreements were settled by discussion or with the assistance of the corresponding author (FY). The overall quality of the evidence generated by the meta-analysis was assessed according to the Grading of Recommendations Assessment, Development and Evaluation (GRADE) methodology. High risk of bias, imprecision, indirectness, heterogeneity and publication bias were assessed using GRADEpro (https://gradepro.org/). Overall, evaluation results were presented as “very low”, “low”, “moderate” or “high” risk, as shown in Supplementary Material S2.

### 2.6 Date synthesis and statistical analysis

The RevMan software (version 5.4; Cochrane, London, United Kingdom) and Stata software (version 14.0; StataCorp, Texas, United States) were used to perform the statistical of standard meta-analyses and draw the funnel plots to evaluate publication bias. Since the results of each study are measured in the same units, the difference in means is expected to have been the measure of effect size for all results. Studies that reported results of lipid profile in mmol/L were converted to mg/dL. The conversion factor was 1 mg/dl = 0.0259 mmol/L for TC, HDL and LDL; and 1 mg/dl = 0.0113 mmol/L for TG. Serum 25(OH)D in nmol/L were converted to ng/mL with 1 ng/ml = 1/2.496 nmol/L. FPG and FI were converted to mg/dL and uU/mL respectively: 1 mg/dl = 1/18 mmol/L, 1 uU/mL = 1/0.319 pmol/L, serum calcium was converted to mg/dL with 1 mmol/L = 0.2495 mg/dl. The effects of VDS were described using the mean difference (MD). Pooled data were presented with 95% confidence interval (CI). The statistical heterogeneity across the included studies was assessed by using the I^2^ value (Nakagawa et al., 2017), with 50% or higher regarded as high. Higher values of the I^2^ statistic indicate increased heterogeneity and range from 0 to 100%. Effects models were selected based on the heterogeneity of the included studies. If I^2^ is greater than 50% and *p* < 0.05, data were pooled using the random-effects model, otherwise the fixed-effects model was used. To interpret the cause of the heterogeneity, we conducted a subgroup analysis according to the dose of VDS (>3000 IU/day, ≤ 3000 IU/day). Sensitivity analysis was planned to determine the robustness and stability of the meta-analysis results by excluding 1) studies with a high risk of bias and 2) numerical outliers. Evidence of publication bias was assessed with the Egger’s test using Stata if ten or more studies were included in each meta-analysis. *p* < 0.05 was considered as statistically significant.

## 3 Results

### 3.1 Search results and study characteristics

**FIGURE 1:**
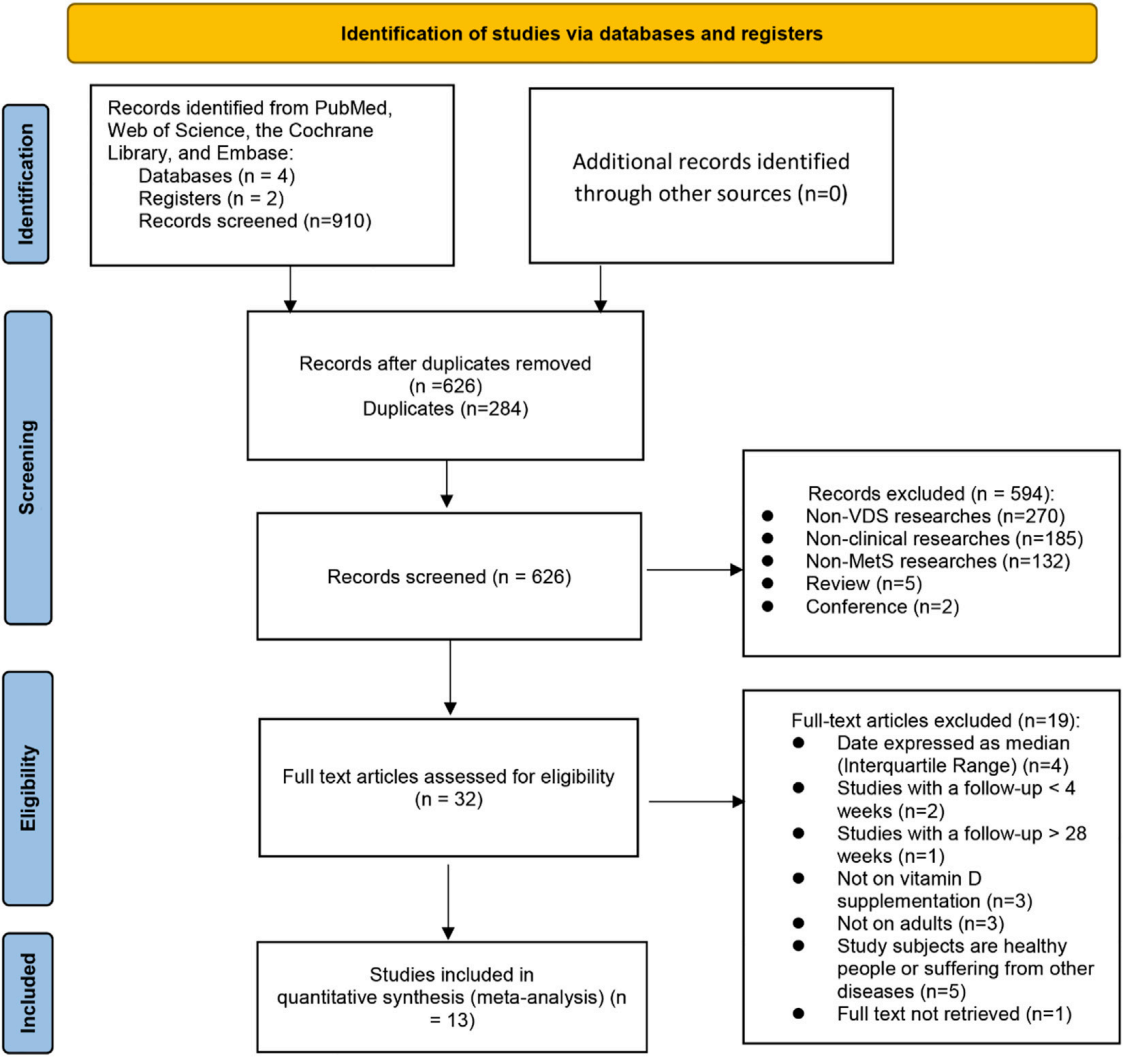
Preferred Reporting Items for Systematic Reviews and Meta-Analyses (PRISMA) diagram of study selection.

The flowchart of search procedure is shown in Figure 1. A total of 910 articles were extracted from four databases, and no further articles were retrieved when reference lists were searched. In all, 626 articles were examined after the removal of duplicates. Based on the titles and abstracts, an additional 594 studies were excluded as they were non-clinical researches (*n* = 185), non-VDS researches (*n* = 270), non-MetS researches (*n* = 132), review (*n* = 5), conference (*n* = 2). There were 32 full text articles left, from which we excluded 19 trials based on 1) date expressed as median (Interquartile range, IQR) (*n* = 4), 2) studies with a follow up less than 4 weeks or longer than 28 weeks (*n* = 3), 3) not on VDS (*n* = 3),4) not on adults (*n* = 3), 5) subjects are healthy people or suffering from other diseases (*n* = 5), 6) full text not retrieved (*n* = 1). Ultimately, a total of 13 RCT articles (Wongwiwatthananukit et al., 2013; Raja-Khan et al., 2014; Mahmood et al., 2016; Salekzamani et al., 2016; Maktabi et al., 2017; Farag et al., 2019; Makariou et al., 2019; Shidfar et al., 2019; Corcoy et al., 2020; Imanparast et al., 2020; Cojic et al., 2021; Ebadi et al., 2021; Pragya et al., 2021) were included in the final systematic review and meta-analysis, for a total of 1,076 study subjects.

The characteristics of 13 included RCTs in the systematic review are presented in Table 1. These studies have investigated 1,076 participants and were published between 2012 and 2022. All of these studies used the RCT design. Among eligible studies, five investigations were conducted in Iran, one in Montenegro, one in Switzerland, one in India, one in Greece, one in the United States, one in Thailand, one in the Netherlands, and one in seven European countries (United Kingdom, Ireland, Austria, Poland, Italy (Padua, Pisa), Spain, and Belgium). Seven of them were reported from Asian countries, and other reports were from non-Asian regions. Ten studies were conducted in developed countries, and others were from developing countries. VDS intervened in different ways, including VD3 or VD2. VDS was also administered at different doses and durations time. Confounding factors including age and gender were controlled in 13 studies.

**TABLE 1:** Information of included studies.

Included studies	Country	NO.(I/C)	Treatment Group	Control Group	Course of Treatment	Daily dose equivalent (IU)	Clinical Outcomes	Criteria for metabolic syndrome
Ebadi et al. (2021)	Iran	32/32	Vitamin D3	Placebo	50,000 IU/week, 8 weeks	7,142.9	①③⑦⑧⑨⑩⑪⑫⑬⑳	_
Cojic et al. (2021)	Montenegro	49/65	Vitamin D3	Placebo	50,000 IU/week during the first 3 months and 14 000 IU/week for the next 3 months	4,571.4	①③④⑤⑦⑧⑨⑩⑫⑬⑮⑯⑰⑳	ADA 2011
Pragya et al. (2021)	India	50/51	Vitamin D3	Placebo	120,000 IU/month, 24 weeks	4,285.7	①③⑥	WHO
Imanparast et al. (2020)	Iran	46/46	Vitamin D3	Placebo	50,000 IU/week, 24 weeks	7,142.9	①④⑤⑦⑧⑨⑩⑪⑫⑬⑮⑱⑲⑳	ADA
Corcoy et al. (2020)	European countries	79/75	Vitamin D3	Placebo	1600 IU/day, 24–28 weeks	1,600.0	⑪⑲⑳	WHO 2013
Makariou et al., 2018	Greece	25/25	Vitamin D3	Placebo	2000 IU/day, 12 weeks	2000.0	①③	NCEP-ATP III
Shidfar et al. (2019)	Iran	37/36	Vitamin D3	Placebo	1000 IU/day, 12 weeks	1,000.0	①②⑥⑳	_
Maktabi et al. (2017)	Iran	35/35	Vitamin D3	Placebo	50,000 IU/2 weeks, 12weeks	3,571.4	⑦⑧⑨⑩⑪⑫⑬⑭⑯⑰⑳	Rotterdam criteria
Salekzamani, et al. (2016)	Iran	35/36	Vitamin D3	Placebo	50,000 IU/week, 16 weeks	7,142.9	①②③④⑤⑥⑦⑧⑨⑩⑫⑬⑭⑳	IDF
Raja-Khan et al. (2014)	United States	13/15	Vitamin D3	Placebo	12,000 IU/week, 12 weeks	1714.3	①④⑤⑦⑧⑨⑩⑪⑫⑬⑭⑰⑱⑳	NIH 1990
Wongwiwatthananukit et al. (2013) (a)	Thailand	28/28	Vitamin D2	Placebo	20,000 IU/week, 8 weeks	2,857.14	⑦⑧⑨⑩⑪⑫⑬⑳	NCEP-ATP III
Wongwiwatthananukit et al. (2013) (b)	Thailand	28/28	Vitamin D2	Placebo	40,000 IU/week, 8 weeks	5,714.28	⑦⑧⑨⑩⑪⑫⑳	NCEP-ATP III
Farag et al. (2019)	Iraq	24/25	Vitamin D	Placebo	2000 IU/day, 12 weeks	2000.0	⑦⑧⑨⑩⑬⑳	IDF
Mahmood et al. (2016)	India	49/49	Vitamin D3	Placebo	60,000 IU/week for 8 weeks followed by 60,000 IU/month for 16 weeks	4,285.7	①③④⑤⑪⑫⑬⑭⑳	IDF

No., number of participants; I, intervention group; C, control group; Outcome Indicators: ① BMI: body mass index; ② WHR: waist to hip ratio; ③ WC: waist circumference; ④ SBP: systolic blood pressure; ⑤ DBP: diastolic blood pressure; ⑥ BF%: body fat percentage; ⑦ TC: total cholesterol; ⑧ TG: triglyceride; ⑨ HDL-C: high-density lipoprotein cholesterol; ⑩ LDL-C: low-density lipoprotein cholesterol; ⑪ FPG: fasting plasma glucose; ⑫ FI: fasting insulin; ⑬ HOMA-IR: homeostasis model assessment of insulin resistance; ⑭ QUICKI: quantitative insulin check index; ⑮ HbA1c: glycosylated hemoglobin, type A1C; ⑯ MDA: malondialdehyde; ⑰ hs-CRP: hypersensitive C-reactive protein; ⑱PTH: parathyroid hormone; ⑲ serum calcium; ⑳ 25(OH)D. Abbreviations: ADA, american diabetes association; WHO, world health organization; NCEP-ATP III, National Cholesterol Education Program’s Adult Treatment Panel III; IDF, international diabetes federation; NIH, national institutes of health.

### 3.2 Assessment of bias risk

**FIGURE 2:**
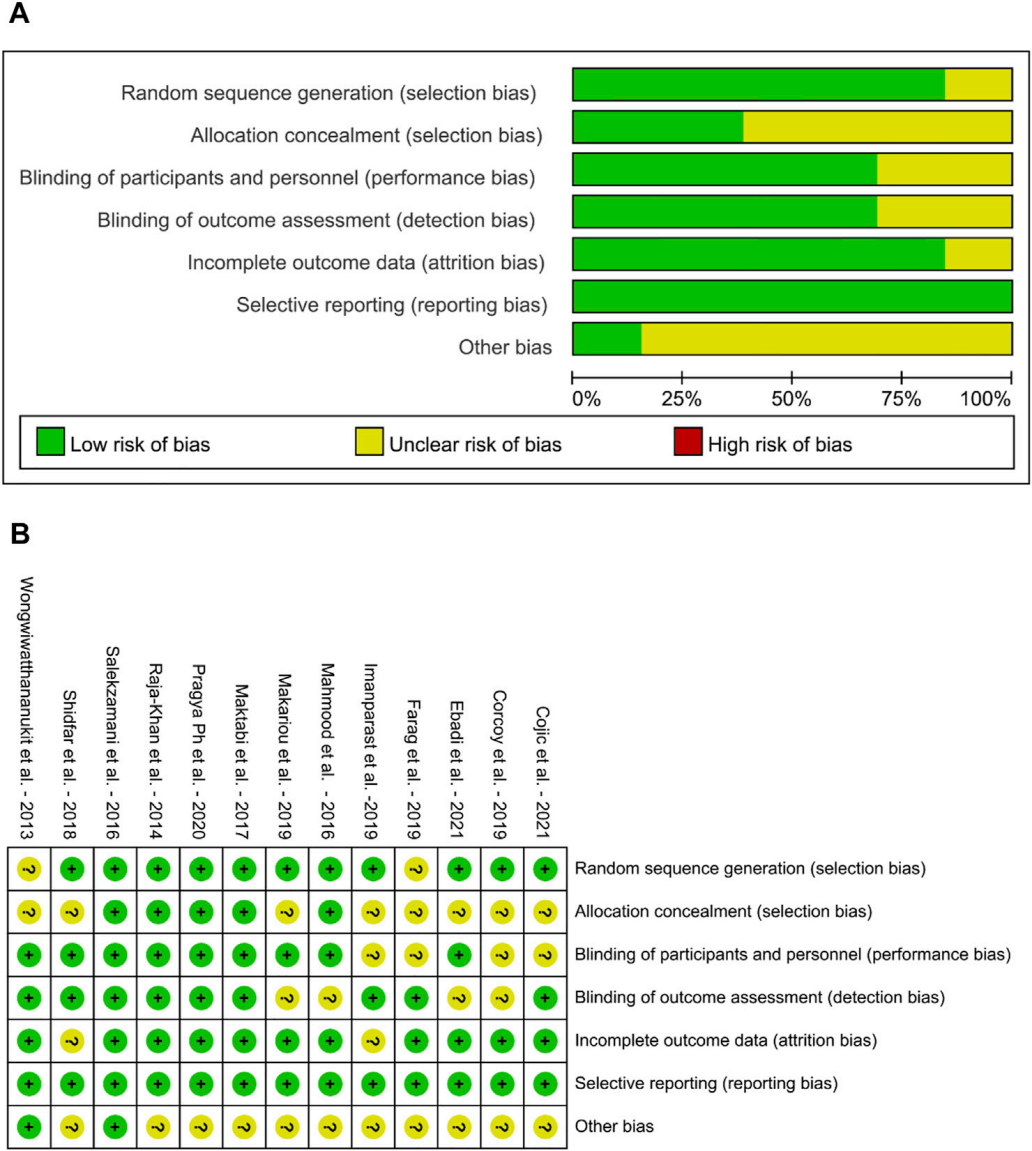
Risk of bias graph of Included Studies **(A)** Risk of bias graph **(B)** Risk of bias summary.

The assessment results of the bias risk in the included RCTs are shown in Figure 2. According to the Cochrane criteria, the overall quality of all RCT design and reporting were good albeit it varied among trials. Individuals were assigned in all investigations by the randomization method, only 2 trails (Wongwiwatthananukit et al., 2013; Farag et al., 2019) were not reported. Five RCTs gave sufficient details to ascertain adequate allocation concealment (Raja-Khan et al., 2014; Mahmood et al., 2016; Salekzamani et al., 2016; Maktabi et al., 2017; Pragya et al., 2021), while this was unclear in the other studies. Blinding of participants was not clear in 4 trails (Farag et al., 2019; Corcoy et al., 2020; Imanparast et al., 2020; Cojic et al., 2021), since they did not mention the randomization method. All trials had adequate blinding of outcome assessment, except 4 trails (Mahmood et al., 2016; Makariou et al., 2019; Corcoy et al., 2020; Ebadi et al., 2021) were unclear. Only 2 trials reported incomplete outcome date (Shidfar et al., 2019; Imanparast et al., 2020), the rest of studies were in low risk of attrition bias. All trails had low selective reporting bias.

## 4 Results of the included RCTs

The outcomes and results from the included studies are described in Table 2. All the included RCTs reported significant increase in serum 25(OH)D levels in the intervention groups at endline. For anthropometric parameters and blood pressure, BMI was analyzed in 9 trails (Raja-Khan et al., 2014; Mahmood et al., 2016; Salekzamani et al., 2016; Makariou et al., 2019; Shidfar et al., 2019; Imanparast et al., 2020; Cojic et al., 2021; Ebadi et al., 2021; Pragya et al., 2021). Eight of them showed no difference in BMI after the intervention compared to baseline values in the experimental group. Only 1 trail showed a significant reduction after VDS treatment (Mahmood et al., 2016). Meanwhile, 2 trails also assessed WHR (Salekzamani et al., 2016; Shidfar et al., 2019). Four trails reported that the VDS intervention had not significant effect on WC except for 1 trail (Mahmood et al., 2016). Regarding end-point values of blood lipid profile, almost all trails have consistently shown that no significant effect can be observed after VDS treatment. Only 1 trail demonstrated that VDS significantly reduced TC and TG levels in patients with MetS (Salekzamani et al., 2016). According to Salekzamani et al., it is worth noting that TG, TC and HDL-C were significantly higher in the intervention group than the control group at baseline (Salekzamani et al., 2016). In the glycemia aspects, 5 trails showed that there was no statistically significant difference in HOMA-IR or any the other outcomes about FPG, FI or QUICKI (Wongwiwatthananukit et al., 2013; Raja-Khan et al., 2014; Salekzamani et al., 2016; Corcoy et al., 2020; Cojic et al., 2021). However, 2 trails reported a greater decrease in FPG, FI and HOMA-IR in the VDS group compared with the placebo group (Maktabi et al., 2017; Ebadi et al., 2021). Regarding biomarkers of inflammation and oxidative stress, Imanparast, F. et al. reported a greater decrease in MDA and hs-CRP after the treatment of VDS (Imanparast et al., 2020), which was not found in the study conducted by Cojic et al. (Cojic et al., 2021).

**TABLE 2:** Outcomes and results of included studies.

First author, year	Baseline 25(OH)D	Endline 25(OH)D	Baseline outcomes	Endline outcomes	Conclusion
Ebadi et al. (2021)	I: 12.2 ± 5.6	I:38.6 ± 8.1	BMI	BMI	High-dose vitamin D3 in obese and overweight individuals with low levels of vitamin D could improve their glycemic status, including FBS, insulin, HOMA-IR. However, no significant reduction was found in the lipid profile
	C: 14.5 ± 6.6	C:14.9 ± 6.4	I:28.0 ± 2.3 C:29.4 ± 3.3	I:28.5 ± 2.4 C:29.0 ± 4.2	
			TC	TC	
			I:188.5 ± 35.5 C:181.5 ± 32.6	I:179.1 ± 33.4 C:182.2 ± 30.4	
			TG	TG	
			I:115.8 ± 53.5 C:124.9 ± 42.0	I:109.8 ± 48.8 C:125.6 ± 39.2	
			HDL-C	HDL-C	
			I:46.2 ± 6.4 C:43.5 ± 8.1	I:46.7 ± 6.9 C:45.6 ± 7.0	
			LDL-C	LDL-C	
			I:116.7 ± 30.3 C:113.6 ± 26.6	I:113.7 ± 29.4 C:117.0 ± 25.3	
			FPG	FPG	
			I:91.9 ± 7.6 C:91.0 ± 6.1	I:85.7 ± 7.6 C:93.1 ± 6.9	
			FI	FI:	
			I:13.9 ± 6.0 C:13.1 ± 3.4	I:8.7 ± 5.1 C:13.5 ± 3.7	
			HOMA-IR	HOMA-IR	
			I:3.1 ± 1.4 C:2.9 ± 0.8	I:1.8 ± 1.1 C:3.2 ± 0.9	
Cojic et al. (2021)	I:19.55 ± 12.67	I:36.96 ± 8.11	BMI	BMI	Study has indicated that daily doses of vitamin D reduce the levels of HbA1c. Its effect on metabolic control through the improvement on HOMA-IR, and oxidative stress
	C:23.24 ± 12.95	C:20.74 ± 9.61	I:30.1 ± 4.6 C:29.8 ± 5.0	I:29.7 ± 7.8 C:28.8 ± 6.1	
			WC	WC	
			I:103 ± 11 C:105 ± 11	I:104 ± 11 C:105 ± 11	
			SBP	SBP	
			I:136.66 ± 24 C:139.48 ± 19.15	I:136.65 ± 17.78C:141.45 ± 16.62	
			DBP	DBP	
			I:83.19 ± 12.14 C:81.28 ± 10.16	I:83.07 ± 8.21 C:84.18 ± 9.67	
			TC	TC	
			I:203.35 ± 74.23C:208.76 ± 63.40	I:221.14 ± 73.45 C:216.50 ± 77.71	
			TG	TG	
			I:152.40 ± 90.37 C:156.82 ± 92.14	I:150.62 ± 95.69C:163.91 ± 124.04	
			HDL-C	HDL-C	
			I:52.19 ± 13.14 C:48.71 ± 11.60	I:53.35 ± 11.60 C:48.33 ± 13.14	
			LDL-C	LDL-C	
			I:129.90 ± 40.98 C:131.83 ± 40.60	I:131.44 ± 40.21 C:124.49 ± 46.01	
			FPG	FPG	
			I:141.66 ± 43.20 C:142.38 ± 25.92	I:130.14 ± 22.68 C:139.32 ± 26.82	
			FI	FI	
			I:11.25 ± 7.43 C:10.66 ± 8.92	I:11.26 ± 6.68 C:11.92 ± 7.86	
			HOMA-IR	HOMA-IR	
			I:3.67 ± 2.63 C:3.64 ± 3.22	I:3.44 ± 2.89 C:3.39 ± 3.37	
			HbA1c	HbA1c	
			I:6.56 ± 1.02 C:6.74 ± 0.81	I:6.48 ± 0.70 C:6.87 ± 0.92	
			MDA	MDA	
			I:3.14 ± 1.89 C:3.31 ± 1.73 hs-CRP:	I:2.77 ± 2.38 C:3.09 ± 3.97 hs-CRP:	
			I:1.79 ± 3.02 C:1.40 ± 2.06	I:1.61 ± 2.93 C:2.13 ± 3.16	
Pragya et al. (2021)			BMI	BMI	There was no statistically significant difference in BMI values or WC, BF% values
			I:31.7 ± 4.1 C:32 ± 3.2	I:31.8 ± 4.6 C:32.0 ± 3.6	
			WC	WC	
			I:98.6 ± 12.1 C:99.1 ± 10.9	I:97.8 ± 12.8 C:97.7 ± 11.5	
			BF%	BF%	
			I:35.6 ± 4.4 C:36.1 ± 5.9	I: 35.9 ± 4.4 C: 36.3 ± 6.5	
Imanparast et al. (2020)	I:17.58 ± 8.86	I:51.79 ± 16.48	BMI	BMI	No significant differences were found in the mean of FBS and HbA1c levels. Serum insulin was not significantly different. The change in the lipid parameters (TC, HDL-C, LDL-C and TG) did not show any significant difference
	C:27.82 ± 19.87	C:29.02 ± 20.68	I: 28.29 ± 2.64 C: 28.38 ± 2.14	I: 28.55 ± 2.73 C: 28.72 ± 2.33	
			SBP	SBP	
			I:131.15 ± 21.40 C: 130.81 ± 22.23	I:129.14 ± 19.32 C:130.63 ± 20.52	
			DBP	DBP	
			I:80.63 ± 11.01 C: 80.43 ± 10.76	I: 77.82 ± 11.78 C: 78.18 ± 11.57	
			TC	TC	
			I: 177.13 ± 46.53 C: 172.33 ± 42.61	I:185.58 ± 48.79 C:180.79 ± 52.21	
			TG	TG	
			I:210.687 ± 107.73 C:241.82 ± 178.61	I:238.24 ± 181.68 C:242.58 ± 232.47	
			HDL-C	HDL-C	
			I:46.67 ± 11.64 C: 42.93 ± 8.51	I: 45.33 ± 11.12 C: 42.28 ± 7.58	
			LDL-C	LDL-C	
			I:90.72 ± 29.92 C: 83.33 ± 26.22	I: 98.93 ± 33.22 C: 85.51 ± 28.12	
			FPG	FPG	
			I:164.87 ± 42.10 C: 170.12 ± 68.33	I:178.09 ± 49.52 C:176.14 ± 74.25	
			FI	FI	
			I: 1.38 ± 5.12 C: 8.24 ± 5.23	I: 8.01 ± 4.42 C: 14.81 ± 8.18	
			HOMA-IR	HOMA-IR	
			I: 2.77 ± 1.63 C: 3.10 ± 2.08	I: 3.38 ± 1.92 C: 1.35 ± 6.22	
			HbA1c	HbA1c	
			I: 8.12 ± 1.43 C: 8.46 ± 2.61	I: 8.38 ± 1.56 C: 8.52 ± 2.09	
			PTH	PTH	
			I: 46.23 ± 39.2 7 C: 34.82 ± 22.89 serum calcium:	I: 40.56 ± 43.2 9 C: 34.52 ± 14.7 6 serum calcium:	
			I: 9.24 ± 0.71 C: 9.17 ± 0.36	I: 9.59 ± 18.18 C: 9.35 ± 0.37	
Corcoy et al. (2020)	I:29.37 ± 10.74	I: 47.84 ± 14.22	FPG	FPG	Low dose VDS had a small effect on FPG.
	C:27.88 ± 10.74	C: 32.81 ± 15.79	I:84.6 ± 5.4 C:84.6 ± 5.4 serum calcium:	I:82.8 ± 7.2 C:84.6 ± 9.0 serum calcium:	
			I: 9.02 ± 0.44 C: 8.94 ± 0.36	I: 8.86 ± 0.40 C: 8.78 ± 0.40	
Makariou et al. (2018)	-	-	BMI	BMI	At endline, serum 25(OH)D level was significantly higher in the I group compared with the C group. There were no significant differences in lipid parameters between groups
			I:32 ± 5.0 C:34 ± 7.6	I:32.4 ± 5.0 C:32.4 ± 5.3	
			WC	WC	
			I:107.7 ± 12.7 C:110 ± 9.0	I:106.3 ± 13.8 C:107.6 ± 9.6	
			TC	TC	
			I: 219 ± 36 C: 231 ± 34	I: 224 ± 37 C: 223 ± 42	
			HDL-C	HDL-C	
			I: 48 ± 10 C: 50 ± 9	I: 49 ± 9 C: 49 ± 10	
			LDL-C	LDL-C	
			I: 140 ± 35 C: 147 ± 26	I: 145 ± 34 C: 152 ± 37	
Shidfar et al. (2019)	I: 9.9 ± 0.64	I: 21.4 ± 0.73	BMI	BMI	There was no statistically significant difference in BMI. However, VDS significantly decreased WHR and BF%
	C: 10 ± 0.63	C: 11 ± 0.78	I: 30.3 ± 0.64 C: 31.3 ± 0.58	I: 29.2 ± 0.67 C: 29.9 ± 0.46	
			WHR	WHR	
			I: 0.97 ± 0.01 C: 0.98 ± 0.01	I: 0.96 ± 0.01 C: 0.97 ± 0.01	
			BF%	BF%	
			I: 35.3 ± 1.2 C: 34.9 ± 1.3	I: 33 ± 1.2 C: 32.6 ± 1.3	
Maktabi et al. (2017)	I:12.8 ± 4.5	I:27.5 ± 9.8	TC	TC	VDS significantly decreased FPG insulin, HOMA-IR, and increased QUICKI. Supplementation with vitamin D also led to significant reductions in serum hs-CRP and plasma MDA levels
	C:14.5 ± 5.1	C:14.4 ± 5.2	I:151.2 ± 22.5 C:160.1 ± 30.4	I:144.5 ± 22.7 C:163.5 ± 32.1	
			TG	TG	
			I:98.4 ± 55.6 C:112 ± 39.3	I:97.8 ± 40.9 C:118.9 ± 48.9	
			HDL-C	HDL-C	
			I:45.4 ± 5.7 C:50.1 ± 8.5	I:45.2 ± 6.4 C:48.9 ± 8.4	
			LDL-C	LDL-C	
			I:86.1 ± 20.9 C:87.6 ± 25.8	I:79.8 ± 26.3 C:90.8 ± 28.7	
			FPG	FPG	
			I:91 ± 6.1 C:93.8 ± 7.8	I:87.8 ± 7.6 C:94.3 ± 9.8	
			FI	FI	
			I:9.6 ± 4.5 C:9.1 ± 7.3	I:8.2 ± 2.8 C:11.7 ± 6.5	
			HOMA-IR	HOMA-IR	
			I:2.2 ± 1.1 C:2.1 ± 1.7	I:1.8 ± 0.6 C:2.7 ± 1.6	
			QUICKI	QUICKI	
			I:0.34 ± 0.02 C:0.36 ± 0.05	I:0.35 ± 0.02 C:0.34 ± 0.04	
			MDA	MDA	
			I:2.2 ± 0.4 C:2.1 ± 0.8 hs-CRP:	I:2.1 ± 0.4 C:3.0 ± 1.7 hs-CRP:	
			I:2.6 ± 2.8 C:2.9 ± 2.5	I:1.9 ± 1.7 C:3.5 ± 2.9	
Salekzamani et al. (2016)	I: 16.45 ± 15.50	I: 78.38 ± 21.71	BMI	BMI	At endline, 25(OH)D significantly increased in the I group and was stable in the C group. TG and TG/HDL-C had a greater change in the I compared with the C group. There were no significant differences in other lipid parameters between groups
	C: 23.47 ± 21.34	C: 21.46 ± 17.74	I:33.17 ± 4.83 C:33.58 ± 4.35	I:33.14 ± 4.97 C:33.49 ± 4.28	
			WHR	WHR	
			I:0.97 ± 0.05 C:0.95 ± 0.07	I:0.96 ± 0.05 C:0.95 ± 0.05	
			WC	WC	
			I:106.7 ± 11.8 C:105 ± 9.7	I:105 ± 11.8 C:105 ± 9.1	
			SBP	SBP	
			I:133 ± 14 C:130 ± 10	I:125 ± 13 C:127 ± 12	
			DBP	DBP	
			I:85 ± 10 C:23 ± 8	I:82 ± 11 C:81 ± 9	
			BF%	BF%	
			I:33.37 ± 6.99 C:32.97 ± 7.36	I:32.97 ± 7.22 C:32.69 ± 7.43	
			TC	TC	
			I:212 ± 42.00 C:200 ± 39.27	I:203.21 ± 34.63 C:197.14 ± 33.57	
			TG	TG	
			I:269 ± 97 C:185 ± 61	I:242 ± 82 C:196 ± 72	
			HDL-C	HDL-C	
			I:45 ± 8.08 C:45 ± 10.08	I:47 ± 6.63 C:47 ± 8.24	
			LDL-C	LDL-C	
			I:114 ± 33 C:117 ± 28	I:106 ± 25 C:111 ± 29	
			FPG	FPG	
			I:93 ± 15 C:95 ± 12	I:96 ± 12 C:95 ± 12	
			FI	FI	
			I:12.05 ± 5.19 C:12.75 ± 4.87	I:11.31 ± 5.76 C:11.99 ± 5.47	
			HOMA-IR	HOMA-IR	
			I:2.76 ± 1.26 C:2.96 ± 1.19	I:2.68 ± 1.38 C:2.76 ± 1.24	
			QUICK	QUICKI
			I:0.33 ± 0.02 C:0.33 ± 0.02	I:0.34 ± 0.03 C:0.33 ± 0.02	
Raja-Khan et al. (2014)	I:19.95 ± 9.47	I:67.36 ± 28.62	BMI	BMI	Compared to placebo, VDS significantly increased serum 25 (OH)D level. There were no significant differences in QUICKI and other measures of insulin sensitivity. There was a protective effect of vitamin D on blood pressure
	C:22.20 ± 6.86	C:22.45 ± 7.02	I:37.2 ± 4.53 C:35.09 ± 9.81	I:37.85 ± 4.50 C:37.69 ± 10.00	
			SBP	SBP	
			I:117.46 ± 10.00 C:113.91 ± 10.21	I:118.56 ± 6.67 C:118.41 ± 10.53	
			DBP	DBP	
			I:79.08 ± 8.28 C:74.88 ± 7.72	I:78.97 ± 2.27 C:80.00 ± 8.31	
			TC	TC	
			I:172.00 ± 42.70 C:184.27 ± 32.52	I:177.18 ± 37.17 C:181.09 ± 40.10	
			TG	TG	
			I:139.08 ± 76.61 C:149.47 ± 85.46	I:127.73 ± 58.09 C:150.73 ± 76.95	
			HDL-C	HDL-C	
			I:45.54 ± 17.60 C:37.00 ± 10.83	I:45.73 ± 18.40 C:37.27 ± 10.73	
			LDL-C	LDL-C	
			I:98.62 ± 36.96 C:117.33 ± 28.56	I:105.91 ± 27.69 C:116.55 ± 32.69	
			FPG	FPG	
			I:84.92 ± 9.46 C:83.73 ± 9.33	I:83.82 ± 8.02 C:77.64 ± 14.66	
			FI	FI	
			I:26.31 ± 9.60 C:27.13 ± 15.79	I:38.09 ± 37.60 C:28.73 ± 14.64	
			HOMA-IR	HOMA-IR	
			I:5.47 ± 1.82 C:5.80 ± 3.90	I:7.79 ± 7.37 C:5.69 ± 2.97	
			QUICKI	QUICKI	
			I:0.302 ± 0.014 C:0.307 ± 0.029 hs-CRP:	I:0.296 ± 0.022 C:0.309 ± 0.039 hs-CRP:	
			I:7.95 ± 5.24 C:4.42 ± 4.34	I:9.13 ± 5.13 C:6.33 ± 7.30	
			PTH	PTH	
			I: 40.81 ± 27.34 C: 33.17 ± 17.73	I: 15.82 ± 12.27 C: 16.83 ± 13.20	
Wongwiwatthananukit et al. (2013)	I(a):15.08 ± 3.16	I(a):26.80 ± 6.37	TC	TC	At endline, serum 25(OH)D was significantly higher in the I(a) and I(b) groups compared with the C group. There were no significant differences in lipid parameters between groups
	I(b):14.29 ± 3.35	I(b):30.03 ± 6.97	I(a):166.89 ± 20.95 I(b):180.36 ± 34.43	I(a):170.54 ± 39.83 I(b):182.04 ± 31	
	C:16.20 ± 2.99	C:18.99 ± 6.71	C:174.29 ± 38.90	C:175.06 ± 39.12	
			TG	TG	
			I(a):132.29 ± 62.36 I(b):139.32 ± 61.26	I(a):137.79 ± 53.48 I(b):144.82 ± 64.07	
			C:129.46 ± 59.75	C:135.75 ± 71.40	
			HDL-C	HDL-C	
			I(a):52.36 ± 11.86 I(b):53.18 ± 12.46	I(a):50.96 ± 12.21 I(b):52.54 ± 13.49	
			C:53.43 ± 12.73	C:53.46 ± 11.75	
			LDL-C	LDL-C	
			I(a):96.68 ± 19.96 I(b):107 ± 27.46	I(a):102.96 ± 35.09 I(b):110.54 ± 27.47	
			C:102.50 ± 29.51	C:105.61 ± 32.31	
			FPG	FPG	
			I(a):112.39 ± 32.47 I(b):122.89 ± 53.28	I(a):113.07 ± 22.53 I(b):126.89 ± 48.21	
			C:113.89 ± 26.74	C:116.14 ± 28.29	
			FI	FI	
			I(a):6.35 ± 4.34 I(b):6.86 ± 4.61	I(a):6.2 ± 4.04 I(b):7.03 ± 5.7	
			C:4.78 ± 3.47	C:5.91 ± 5.29	
			HOMA-IR	HOMA-IR	
			I(a):1.66 ± 1.03 I(b):2.05 ± 1.55	I(a):1.65 ± 1.01 I(b):2.34 ± 2.29	
			C:1.32 ± 1.03	C:1.76 ± 1.70	
Farag et al. (2019)	I: 26.70 ± 6.98	I: 57.90 ± 12.23	TC	TC	At endline, serum 25(OH)D was significantly higher in the I group compared with the C group. TC, LDL-C were significantly decreased in the I group. There were no significant differences in TG and HDL-C between baseline and endline
	C: 30.20 ± 9.73	C: 31.44 ± 9.98	I: 173.5 ± 60.8 C: 185.9 ± 39	I: 160.5 ± 33.4 C: 196.8 ± 39.4	
			HDL-C	HDL-C	
			I: 34.9 ± 17.3 C: 30.04 ± 8.5	I: 33.7 ± 10.6 C: 31.8 ± 7.0	
			LDL-C	LDL-C	
			I: 120.7 ± 64.4 C: 150.4 ± 39.8	I: 107 ± 36.6 C: 158.8 ± 39	
			TG	TG	
			I: 229.3 ± 113.8 C: 174.4 ± 43	I: 233.8 ± 97 C: 158.6 ± 35.4	
Mahmood et al. (2016)	I: 15.4 ± 9.03	I: 26.1 ± 11.8	SBP	SBP	VDS did not show any significant effect on fasting glucose insulin or insulin resistance indices. There was a significant decrease in BMI and WC. Vitamin D levels increased significantly
	C: 13.3 ± 7.91	C: 13.3 ± 7.14	I: 134 ± 14.9 C: 128 ± 11.4	I: 131 ± 14.6 C: 130 ± 12.2	
			DBP	DBP	
			I: 88.1 ± 9.17 C: 84.8 ± 6.73	I: 85.9 ± 8.35 C: 84.7 ± 8.27	
			BMI	BMI	
			I: 29.1 ± 4.06 C: 29.7 ± 4.44	I: 28.5 ± 4.16 C: 29.5 ± 4.53	
			WC	WC	
			I: 95.9 ± 6.66 C: 96.0 ± 8.07	I: 94.6 ± 7.47 C: 95.5 ± 8.02	
			FI	FI	
			I: 10.8 ± 5.14 C: 10.7 ± 4.81	I: 13.3 ± 8.19 C: 15.4 ± 14.0	
			QUICKI	QUICKI	
			I: 0.33 ± 0.02 C: 0.34 ± 0.03	I: 0.32 ± 0.03 C: 0.32 ± 0.03	
			FPG	FPG	
			I: 103 ± 15.9 C: 103 ± 24.8	I: 103 ± 20.7 C: 103 ± 20.7	
			HOMA	HOMA	
			I: 2.72 ± 1.33 C: 2.82 ± 2.01	I: 3.35 ± 2.09 C: 3.94 ± 3.55	

No., number of participants; I, interventionn group; C, control group; Outcome Indicators: BMI, body mass index; WHR, waist to hip ratio; WC, waist circumference; SBP, systolic blood pressure; DBP, diastolic blood pressure; BF%, body fat percentage; TC, total cholesterol; TG, triglyceride; HDL-C, high-density lipoprotein cholesterol; LDL-C, low-density lipoprotein cholesterol; FPG, fasting plasma glucose; FI, fasting insulin; HOMA-IR, homeostasis model assessment of insulin resistance; QUICKI, quantitative insulin check index; HbA1c, glycosylated hemoglobin, type A1C; MDA, malondialdehyde; hs-CRP, hypersensitive C-reactive protein; PTH, parathyroid hormone. Unified unit: 25(OH)D: ng/mL, WC: cm, SBP: mm/Hg, DBP: mm/Hg, HDL-C, LDL-C, TC, TG: mg/dL; FPG: mg/dL, FI: μU/mL, MDA: μmol/L, hs-CRP: mg/L, PTH: pg/mL, serum calcium: mg/dL.

### 4.1 Results of the meta-analyses

#### 4.1.1 Effect on serum 25(OH)D level

**FIGURE 3:**
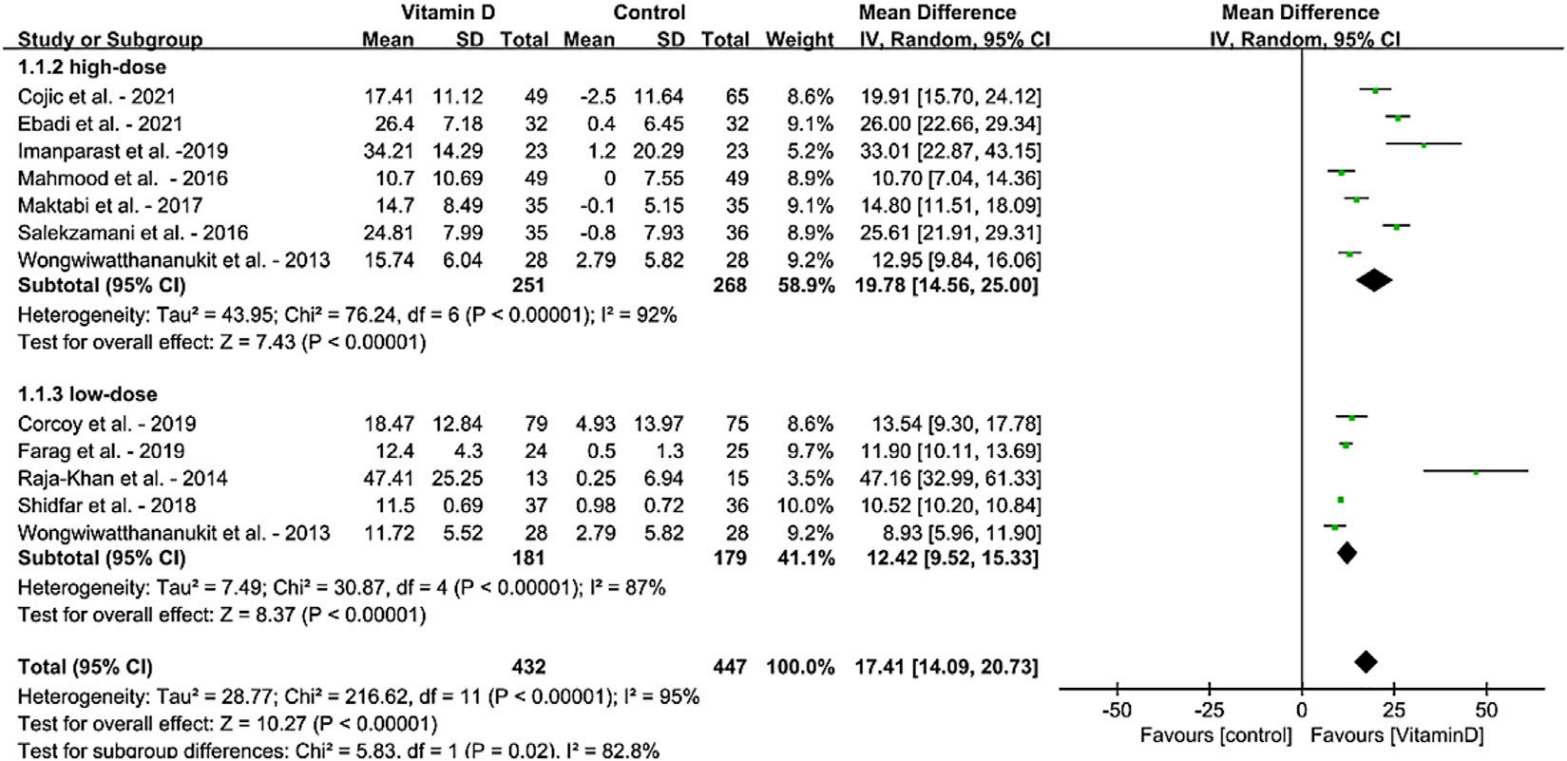
Forest plot for the effects of VDS on serum 25(OH)D levels in MetS adults. The horizontal bar indicates the 95% confidence interval (CI). The size of the rectangle at the center of the horizontal bar is proportional to the weight of the given study. The diamond at the bottom indicates the pooled mean difference (MD).

For the meta-analysis based on the daily vitamin D intervention, the results of forest plots for the MD in serum 25(OH)D levels in MetS adults from 12 RCTs (N = 879) are shown in Figure 3. MD for RCTs is shown as squares, and 95% CIs are indicated by lines across the squares. Diamonds reflect the pooled MDs. When the meta-analysis was conducted, the treatment of VDS significantly increased the overall effects on serum 25(OH)D concentrations [MD:17.41, 95% CI (14.09, 20.73), *p* < 0.00001]. When different doses were considered, the results also showed significant increases in low dose VDS intervention (≤3000 IU/day, 5 trails) [MD: 12.42, 95% CI (9.52, 15.33), *p* < 0.00001] and high dose VDS intervention (>3000 IU/day, 7 trails) [MD: 19.78, 95% CI (14.56, 25.00), *p* < 0.00001].

#### 4.1.2 Effect on anthropometric parameters and blood pressure

**FIGURE 4:**
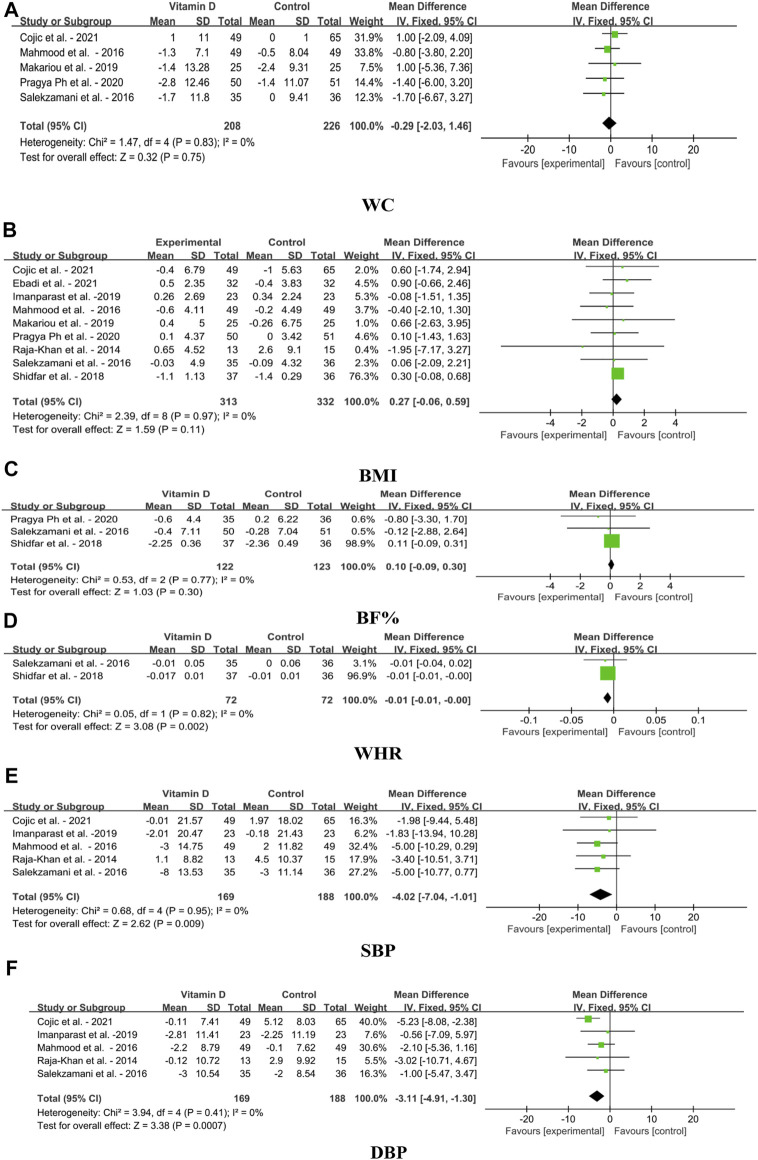
Forest plots for the effects of VDS on anthropometric parameters and blood pressure in MetS adults. **(A)** WC: waist circumference, **(B)** BMI: body mass index, **(C)** BF%: body fat percentage, **(D)** WHR: waist to hip ratio, **(E)** SBP: systolic blood pressure, **(F)** DBP: diastolic blood pressure. The horizontal bar indicates the 95% confidence interval (CI). The size of the rectangle at the center of the horizontal bar is proportional to the weight of the given study. The diamond at the bottom indicates the pooled mean difference (MD).

The effects of VDS on anthropometric parameters and blood pressure in MetS adults include WC, BMI, BF%, WHR, SBP and DBP, as shown in Figures 4A–F The finding showed that VDS had no significant effect on WC [MD: 0.29, 95% CI (−2.03, 1.46), *p* = 0.75], BMI [MD: 0.27, 95% CI (−0.06, 0.59), *p* = 0.11] and BF% [MD: 0.10, 95% CI (−0.09, 0.30), *p* = 0.30]. WHR was slightly decreased [MD: 0.01, 95% CI (−0.01, 0.00), *p* = 0.002] compared with to the control. However, the SBP was significantly decreased after the treatment of VDS [MD: 4.02, 95% CI (−7.04, −1.01), *p* = 0.009], and DBP was also significantly reduced in group receiving VDS [MD: 3.11, 95% CI (−4.91, −1.30), *p* = 0.0007].

#### 4.1.3 Effect on blood lipid profile

**FIGURE 5:**
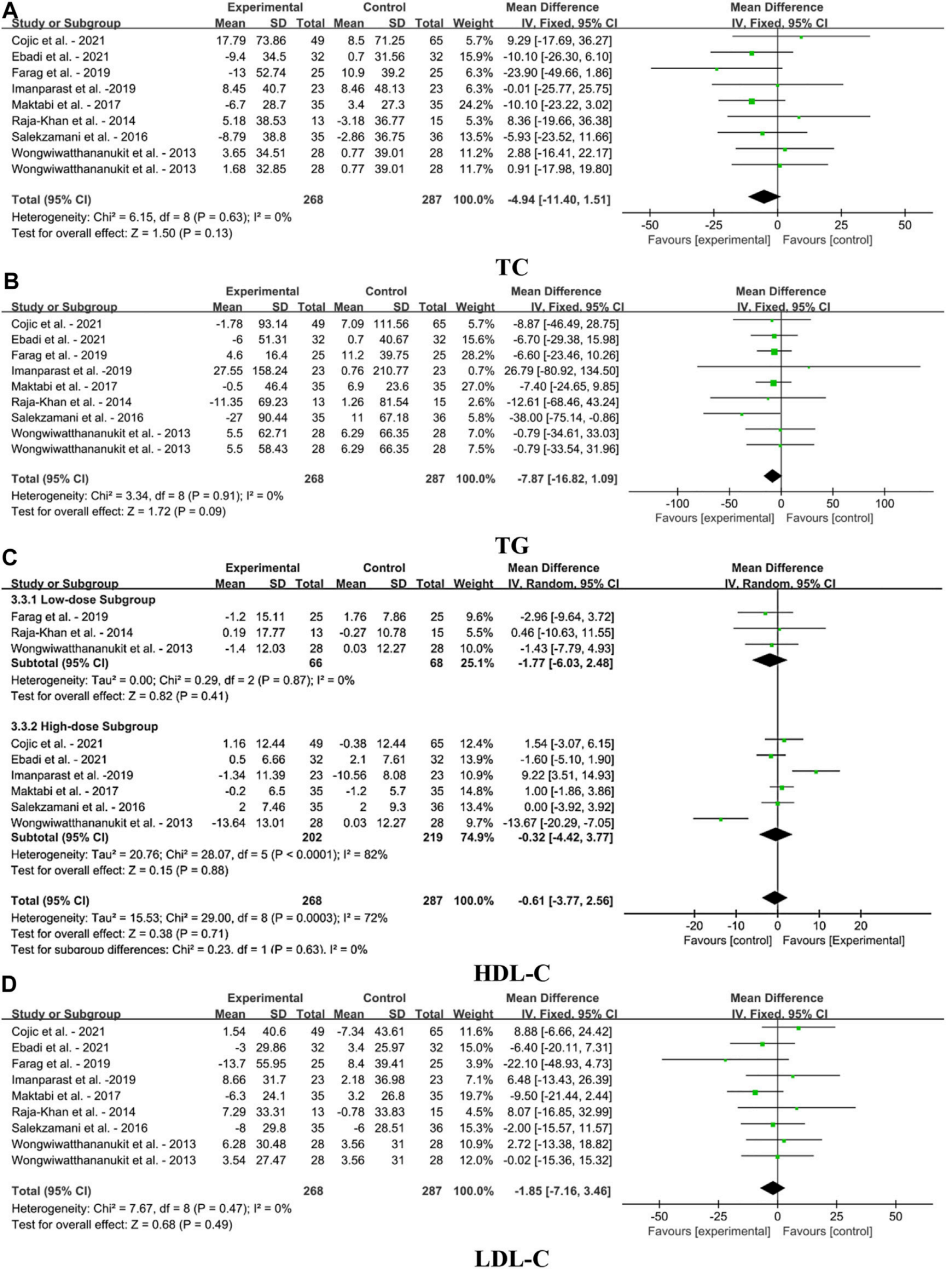
Forest plots for the effects of VDS on serum lipid profile in MetS adults. **(A)** TC: total cholesterol, **(B)** TG: triglyceride, **(C)** HDL-C: high-density lipoprotein cholesterol, **(D)** LDL-C: low-density lipoprotein cholesterol. The horizontal bar indicates the 95% confidence interval (CI). The size of the rectangle at the center of the horizontal bar is proportional to the weight of the given study. The diamond at the bottom indicates the pooled mean difference (MD).

Figures 5A–D illustrates forest plots for the MD in TC, TG, HDL-C and LDL-C for meta-analysis after the VDS intervention on blood lipid profile in MetS adults. The result showed that VDS intervention had no significant effect on TC [MD: 4.94, 95% CI (−11.40, 1.51), *p* = 0.13], TG [MD: 7.87, 95% CI (−16,82, 1.09), *p* = 0.09] and LDL-C concentrations [MD: 1.85, 95% CI (−7.16, 3.46), *p* = 0.49] MetS adults. For HDL-C concentrations, the meta-analysis also revealed that VDS had no significant difference in high-dose subgroup [MD: 0.32, 95% CI (−4.42, 3.77), *p* = 0.88], low-dose subgroup [MD: 1.77, 95% CI (−6.03, 2,48), *p* = 0.41] and overall effects [MD: 0.61, 95% CI (−3.77, 2.56), *p* = 0.71].

#### 4.1.4 Effect on blood glycemic indices

**FIGURE 6:**
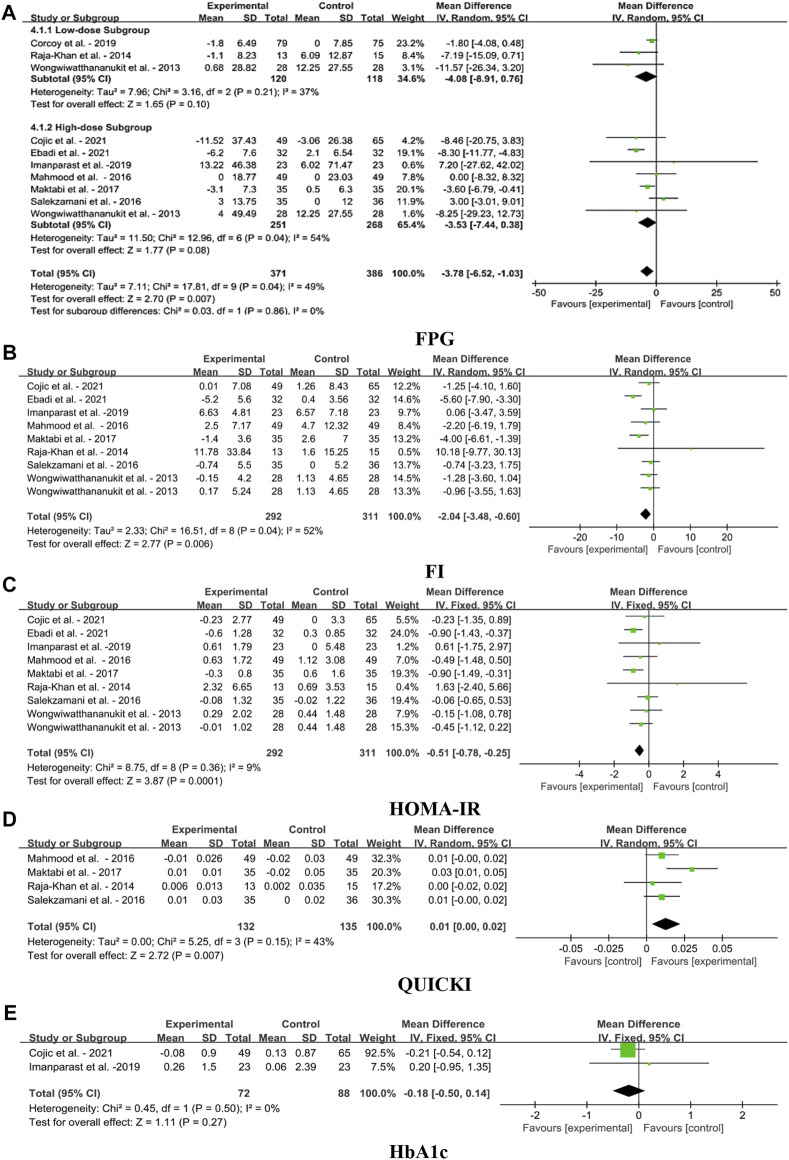
Forest plots for the effects of VDS on serum glycemia aspect in MetS adults. **(A)** FPG: fasting plasma glucose, **(B)** FI: fasting insulin, **(C)** HOMA-IR: homeostasis model assessment of insulin resistance, **(D)** QUICKI: quantitative insulin check index, **(E)** HbA1c: glycosylated hemoglobin, type A1C. The horizontal bar indicates the 95% confidence interval (CI). The size of the rectangle at the center of the horizontal bar is proportional to the weight of the given study. The diamond at the bottom indicates the pooled mean difference (MD).

The forest plots for the MD in FPG, FI, HOMA-IR, QUICKI, and HbA1c levels in MetS adults for meta-analysis based on the VDS intervention as shown in Figures 6A–E. VDS significantly decreased FPG levels [MD: 3.78; 95% CI (−6.52, −1.03), *p* = 0.007], yet the heterogeneity of this parameter was moderate (I^2^ = 49%). Pooled estimate of RCTs indicated significant reduction of FI [MD: 2.04, 95% CI (-3.48, -0.60), *p* = 0.006], and HOMA-IR levels [MD: 0.51, 95% CI (−0.78, −0.25), *p* = 0.0001] after the treatment of VDS. The QUICKI levels were slightly increased the overall effect [MD: 0.01, 95% CI (0.00, 0.02), *p* = 0.007], yet the heterogeneity of this parameter was moderate (I^2^ = 43%). The meta-analysis also showed that VDS had no significant effects on HbA1c levels [MD: 0.18, 95% CI (-0.05, 0.14), *p* = 0.27].

#### 4.1.5 Effect on oxidative stress parameters

**FIGURE 7:**
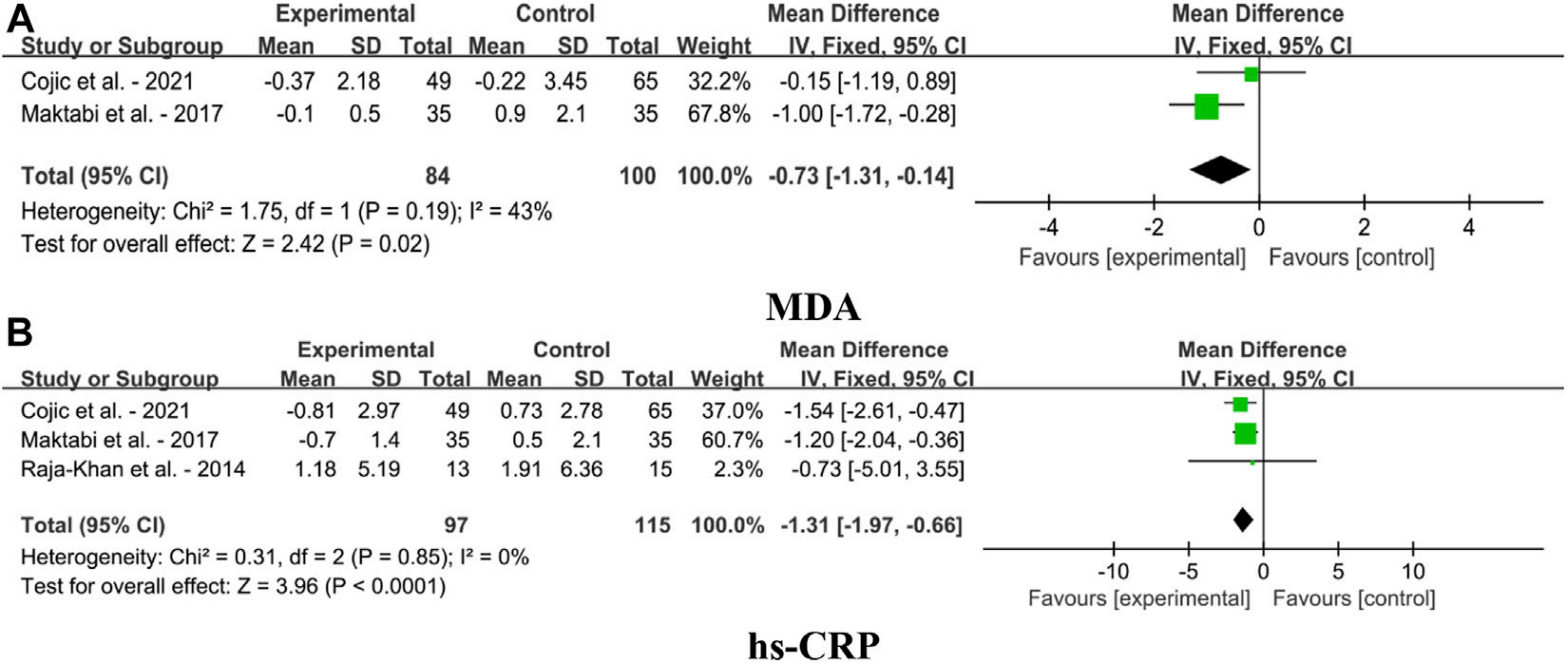
Forest plots for the effects of VDS on oxidative stress parameters in MetS adults. **(A)** MDA: malondialdehyde, **(B)** hs-CRP: hypersensitive C-reactive protein. The horizontal bar indicates the 95% confidence interval (CI). The size of the rectangle at the center of the horizontal bar is proportional to the weight of the given study. The diamond at the bottom indicates the pooled mean difference (MD).

Forest plots for the effects of VDS on oxidative stress parameters in MetS adults, as shown in Figures 7A,B. The findings indicated that VDS significantly lowered the levels of MDA [MD: 0.73, 95% CI (-1.31, -0.14), *p* = 0.02, I^2^ = 43%] and hs-CRP [MD: 1.31, 95% CI (-1.97, -0.66), *p* ＜ 0.0001, I^2^ = 0%].

#### 4.1.6 Effect on VDT biomarkers

**FIGURE 8:**
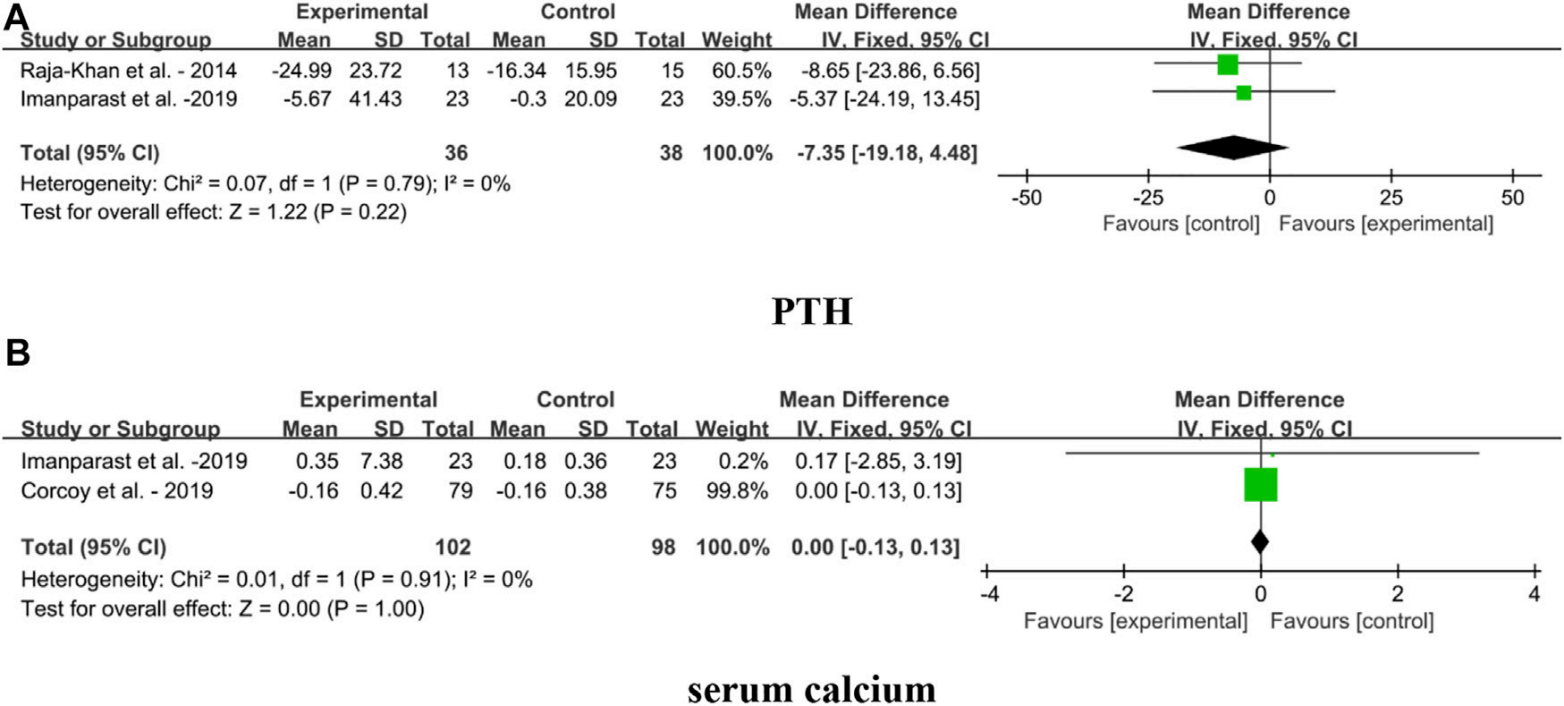
Forest plots for the effects of VDS on VDT biomarkers in MetS adults. **(A)** PTH: parathyroid hormone, **(B)** serum calcium. The horizontal bar indicates the 95% confidence interval (CI). The size of the rectangle at the center of the horizontal bar is proportional to the weight of the given study. The diamond at the bottom indicates the pooled mean difference (MD).

Forest plots for the effects of VDS on VDT biomarkers in MetS adults, including PTH and serum calcium, are shown in Figures 8A,B. The result showed that VDS intervention had no significant effect on PTH [MD: 7.35, 95% CI (−19.18, 4.48), *p* = 0.22, I^2^ = 0%] and serum calcium [MD: 0.00, 95% CI (−0.13, 0.13), *p* = 1.00, I^2^ = 0%].

### 4.2 Publication bias

**FIGURE 9:**
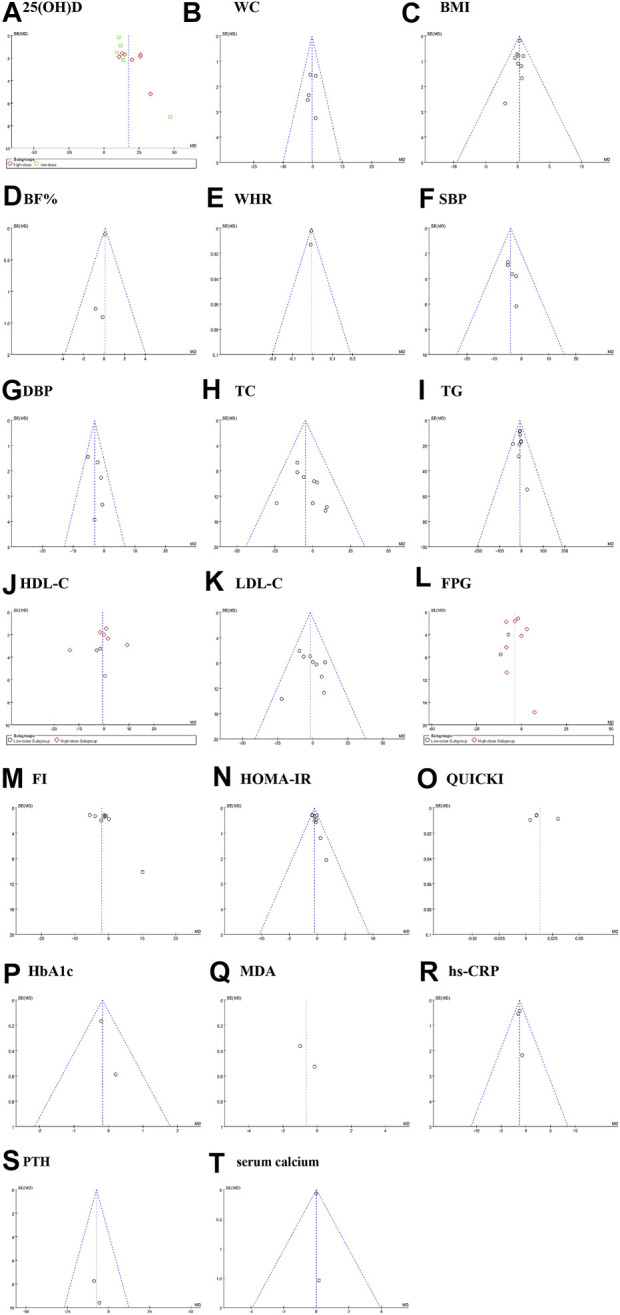
Sensitivity analysis plot. **(A)**: 25(OH)D, **(B)** WC: waist circumference; **(C)** BMI: body mass index; **(D)** BF%: body fat percentage; **(E)** WHR: waist to hip ratio; **(F)** SBP: systolic blood pressure; **(G)** DBP: diastolic blood pressure; **(H)** TC: total cholesterol; **(I)** TG: triglyceride; (**J)** HDL-C: high-density lipoprotein cholesterol; **(K)** LDL-C: low-density lipoprotein cholesterol;**(L)** FPG: fasting plasma glucose;**(M)** FI: fasting insulin;**(N)** HOMA-IR: homeostasis model assessment of insulin resistance;**(O)** QUICKI: quantitative insulin check index;**(P)** HbA1c: glycosylated hemoglobin, type A1C;**(Q)** MDA: malondialdehyde;**(R)** hs-CRP: hypersensitive C-reactive protein. **(S)** PTH: parathyroid hormone; **(T)** serum calcium. Fixed effects model: **(B–k,N,P,R,S,T)**. Random effects model: **(A,L,M,O,Q)**.

Publication bias was assessed by funnel plots. The funnel plots for 25(OH)D, SBP, DBP, BF%, TG, FI and HOMA-IR indicate that there is a possible publication bias, though minimal. However, there were no more than 10 papers available for each meta-analysis, which may be insufficient to identify publication bias using funnel plots (Figure 9).

## 4 Discussion

MetS is a complicated condition characterized by several interrelated risk factors for CVDs and T2DM (Kassi et al., 2011), or even non-alcoholic fatty liver disease (NAFLD) (Tarantino et al., 2019). It is predisposed by atherogenic dyslipidemia (higher TG and apolipoprotein B, small LDL particles, and low HDL-C concentrations), high blood pressure, increased plasma glucose, a prothrombotic condition, and a proinflammatory state (Grundy et al., 2006). Each MetS component may has the potential to impact the endothelium, leading vascular dysfunctions and disturbing vascular homeostasis (Tran et al., 2020). MetS prevalence has been continuously increasing over the last 50 years, thus we need to pay attention to this condition. In parallel, vitamin D insufficiency, which affects about 50% of the world’s population, is increasingly acknowledged as a global health issue (Melguizo-Rodríguez et al., 2021). It is common even in sunny countries (Mendes et al., 2020) and countries that have implemented strict VDS strategies for many years (Ostman et al., 2017). It is generally recognized that inadequate vitamin D levels are related to less sun exposure and deficiency in natural dietary sources (Chang and Lee, 2019). Related studies have shown that vitamin D insufficiency is often present in MetS patients (Obispo Entrenas et al., 2017). However, whether appropriate VDS improves the indicators associated with MetS is still an open question. This research is the first to comprehensively examine and meta-analyze double-blind RCT data on the impact of VDS on a variety of anthropometric and biochemical parameters in MetS patients. The current meta-analysis is the most comprehensive, including 18 MetS component indices and 2 biomarkers of VDT.

The strength of our meta-analysis of RCTs includes its comprehensive indicators in adults with MetS, using a thorough and sensitive search strategy across multiple databases, and explicit date extraction techniques. The results of our study indicated that VDS significantly increased the serum 25(OH)D levels of MetS adults, whether in the form of D2 or D3, in a high or low dose, for a short or long period of time. This finding consistent with the results of previous meta-analysis (Lagowska et al., 2018; AlAnouti et al., 2020). Our meta-analysis of RCTs showed that VDS had no statistically significant effect on anthropometric markers including BMI, WC, and BF% among MetS adults. In line with our study, Sadiya et al. found that VDS had no significant change in BMI, WC and body mass through a cohort study for 6 months on a maintenance dose (Sadiya et al., 2016). A meta-analysis in 2015 conducted by Chandler et al. indicated that VDS had no significant effect on BMI, or BF% when compared with placebo (Chandler et al., 2015). Another meta-analysis performed by Mora et al. also found that oral 25(OH)D supplementation does not significantly impact BMI change (Mora et al., 2013). In contrast, Sharifi et al. reported that the intervention with 50,000 IU VD3 for 4 months had significantly lower BMI and WC compared with the controls in patients with NAFLD (Sharifi et al., 2016). According to Hosseini et al., the intervention group receiving 600,000 IU VD3 also had a substantial decline in BMI and WC of women with NAFLD (Hosseini et al., 2018). Regarding blood pressure, the results of this study indicated that the SBP and DBP were decreased significantly after VDS treatment in patients with MetS. This finding is inconsistent with that of Hussin et al. (Hussin et al., 2017) and Swart et al. (Swart et al., 2018), who observed that VDS did not significantly changes in SBP and DBP. In a study conducted by Scragg R. et al., it was shown that VDS had no effect on DBP or SBP, which is also inconsistent with our findings (Scragg et al., 2014). According to Kamińska et al., the obese state and vitamin D dose may influence the relationship between VDS and metabolic abnormalities. In obese individuals, increasing vitamin D consumption may lower blood pressure and raise HDL-C concentration (Kamińska et al., 2020).

Regarding to lipid profile, our results found that VDS did not affect TC, TG, HDL-C and LDL-C levels in adults with MetS. Another meta-analysis indicated that there were no statistically significant effects of VDS on TC, TG and HDL-C levels in adults (Wang et al., 2012). Interestingly, a meta-analysis performed by AlAnouti et al. found that VDS did not significantly affect TC, HDL-C and LDL-C levels, but significantly increased serum TG levels (AlAnouti et al., 2020). The difference in the results of the effect of vitamin D on serum TG level may explained by the dose, frequency and duration time of intervention. The mean endline serum 25(OH)D levels in the intervention groups of the RCTs included in this study were below the recommended range of 100–150 nmol/L for the prevention of CVDs (Lugg et al., 2015). The underlying mechanisms of VDS on lipid profile remains unclear. To achieve consistent circulation concentrations for the endocrine system’s optimum performance, VDS should be given daily with an appropriate amount (Hollis and Wagner, 2013). Therefore, short treatment times and bolus dosages in some included RCTs may account for the lack of benefits. It is also conceivable that vitamin D may enhance indicators other than the lipid profile, such as endothelial function, or that it might have a positive impact on the serum calcium profile early in the course of the illness (Tabrizi et al., 2018). It has been suggested that the link between vitamin D and MetS may be confused with obesity, not causation. Obese persons may have low levels of 25(OH)D in their blood owing to adipose tissue impeding vitamin D absorption and usage or because they spend less time outside, which leads to insufficient vitamin D production in the skin (Vimaleswaran et al., 2013). In conclusion, there may not be a direct causative relationship between the VDS and lipid status. In other words, high levels of serum 25(OH)D may not be the cause of health, but the consequence. This can be explained by the fact that people who are in good health are more likely to be active in the outdoors and have healthier eating habits (Challoumas, 2014).

This meta-analysis of RCTs showed that VDS had a benefit effect on FPG, FI and HOMA-IR levels in MetS adults, but had slight or no influence on QUICKI and HbA1c levels. Nevertheless, the results of current studies were contradictory. Some studies on pre-diabetic patients (Lemieux et al., 2019; Zhang et al., 2021) and T2DM patients (Farrokhian et al., 2017; Safarpour et al., 2020) have shown the benefits of VDS. A meta-analysis conducted by Zhang et al. reported that VDS resulted in a vast improvement in FPG, HbA1c, and FI levels in prediabetics, but not in other parameters (Zhang et al., 2021). However, a meta-analysis of 23 RCTs investigating the influence of vitamin D on glycemic control in individuals with T2DM revealed no effect on FPG, HOMA-IR and HbA1c (Krul-Poel et al., 2017). A meta-analysis of 10 RCTs conducted by Poolsup et al. showed that no beneficial effect of vitamin D in improving insulin resistance was identified (Poolsup et al., 2016). Another meta-analysis by Li et al. observed no benefit of VDS in improving FPG, HbA1c, and FI in T2DM patients (Li et al., 2018). It is unclear exactly how the low vitamin D levels and poor glucose tolerance are related. The effects are minimal in a population that is already qualified for VDS, and just a few studies have shown therapeutic relevance. Effects may vary depending on ethnicity and genetic make-up. In additional, our meta-analysis observed that VDS considerably reduced serum MDA and hs-CRP levels among MetS adults, which are consistent with other studies (Mansournia et al., 2018; Zhao et al., 2021). However, some opposite results were presented in observational and interventional studies, which indicated that there were no significant changes in hs-CRP in women with polycystic ovary syndrome (Rahimi-Ardabili et al., 2013; Javed et al., 2019). By controlling insulin resistance and/or pancreatic *β*-cell activity, vitamin D may help alleviate the pathogenesis of MetS. Through immunomodulatory effects, vitamin D status or components necessary for its activation or transport may also play a role in the development of type 1 diabetes (Charoenngam and Holick, 2020). These findings led to speculation about a possible connection between vitamin D and diabetes. Nevertheless, bigger RCTs are still required to validate the mechanism of action of VDS on glycemic and oxidative stress parameters.

NAFLD that refers to the exclusion of excessive alcohol consumption and the elevation of hepatic fat deposition affects around 30% of the adult population worldwide (Targher et al., 2021). It includes a series of disease processes. Nonalcoholic fatty liver can progress to nonalcoholic steatohepatitis (NASH), gradually causes liver fibrosis, and may eventually develop into liver cirrhosis, liver failure and even hepatocellular carcinoma (HCC) (Tarantino et al., 2019). Globally, NAFLD cases are projected to expand from 83.1 million in 2015 to 100.9 million in 2030 (Carr et al., 2016), which is highest in the Middle East and South America and lowest in Africa (Younossi et al., 2016). NAFLD is associated with T2DM, CVDs, chronic kidney disease, and certain types of extrahepatic complications (Targher et al., 2021). In 2020, a panel of international experts from 22 countries advocated changing the name of NAFLD to metabolic dysfunction associated fatty liver disease (MAFLD) to underline the systemic aspect of NAFLD and its strong connections to other metabolic diseases (Eslam et al., 2020). An RCT was conducted by Javed et al., and its results showed that VDS women with polycystic ovary syndrome had a significant decrease in ALT (*p* = 0.042) and a weak reduction in HOMA-IR (*p* = 0.051). Hormones, liver indicators, or other cardiovascular risk factors did not show any between-group variations in this RCT (Javed et al., 2019). In the 13 studies included in our meta-analysis, one study (Shidfar et al., 2019) considered metabolic markers of NAFLD, including alanine amino-transferase (ALT) and aspartate aminotransferase (AST). According to the Shidfar et al., individuals with NAFLD may have lower blood atherogenic indices, liver function tests, and disease severity after supplementation of calcium and vitamin D, but not vitamin D only (Shidfar et al., 2019). So, there is no way to do quantitative analysis in our meta-analysis and come to a conclusion to support an association between VDS and NAFLD. However, this does not exclude a certain link between them when more studies are conducted, and more data are analyzed. Future RCTs should include more indicators of NAFLD for correlation analysis such as AST, ALT, hyaluronic acid, creatinine, leptin, adiponectin, resistin, ghrelin, N-terminal pro-peptide of type III procollagen, fibroblast growth factor 21 and retinol binding protein 4, tissue inhibitor of metallo-proteinases-1, plasma cytokeratin 18 and enhanced liver fibrosis score.

Long-term excessive intake of vitamin D may cause toxicity. VDT includes acute toxicity and chronic toxicity. Serum 25(OH)D concentrations >150 ng/ml (>375 nmol/L) with dosages of vitamin D above 10,000 IU/day would likely result in acute toxicity (Marcinowska-Suchowierska et al., 2018). Clearly, this quantity exceeds the IOM-recommended maximum daily intake of 4,000 IU. Serum 25(OH)D values in the range of 50–150 ng/ml (125–375 nmol/L) are associated with a risk of chronic toxicity if administered at dosages above 4,000 IU/day for protracted durations (Marcinowska-Suchowierska et al., 2018). The most often seen clinical manifestations of VDT are confusion, apathy, repeated vomiting, stomach discomfort, polyuria, polydipsia, and dehydration. Exogenous VDT due to excessive vitamin D intake is identified by considerably high 25(OH)D concentrations (>150 ng/ml), severe hypercalcemia and hypercalciuria, and extremely low or undetectable PTH activity (Holick, 2015). Our meta-analysis indicated that there were no statistically significant effects of VDS on PTH and serum calcium levels. Of the 13 included studies, 7 RCTs had an average daily dose of VDS over 4,000 IU (Wongwiwatthananukit et al., 2013; Mahmood et al., 2016; Salekzamani et al., 2016; Imanparast et al., 2020; Cojic et al., 2021; Ebadi et al., 2021; Pragya et al., 2021), and 4 RCTs had the serum 25(OH)D levels over 50 ng/day (Raja-Khan et al., 2014; Salekzamani et al., 2016; Farag et al., 2019; Imanparast et al., 2020). Chronic toxicity of VDT is likely to occur at such vitamin D intake dosages if the duration period lasts more than 7 months (Marcinowska-Suchowierska et al., 2018). Data from a prospective study performed by Misgar et al. showed that VDT leads to symptomatic hypercalcemia. According to this research, VDT should be taken into account in patients, particularly the elderly, who present with polyuria, polydisplasia, vomiting, azotemia, or encephalopathy (Misgar et al., 2019). The key to preventing VDT is to raise general practitioners’ awareness of the toxicity brought on by excessive dosages of vitamin D. If individuals have symptoms of VDT, they must seek medical attention in time, and a low-calcium diet should be adopted. In addition, consumption large quantity of water to promote calcium excretion, and avoidance of sunlight exposure will also help to relieve the symptoms. Patients and prescribers should be more aware of the possible risks of VDT despite its rarity (Galior et al., 2018). We also suggest that future large prospective studies and RCTs can focus on the potential detrimental effects of vitamin D overdose. It is important to include relative indicators including cumulative vitamin D dose, serum calcium, serum phosphate, serum 25(OH)D, serum PTH, and serum creatinine.

Nevertheless, this meta-analysis also has some limitations. First of all, the majority of RCT trials had very small sample sizes, which might result in type-2 statistical error. Second, some research published in non-peer-reviewed documents and publications, that may have been overlooked during the literature search. Third, the majority of the included RCTs in this meta-analysis were conducted in Iran, where women’s skin is often covered by clothes, which blocked sunlight from promoting vitamin D synthesis in the skin. Therefore, it may be inaccurate to presume that the conclusions of these Iranian studies can be directly compared to those of investigations conducted by other countries. Furthermore, we did not take into account this confounding factor due to the small number of studies examining the impact of carrier substances (such as powder or ethanol carriers) on the bioavailability of vitamin D. More extensive prospective studies need to be performed to investigate the bioavailability of various vitamin D carrier compounds in the future.

## 5 Conclusion

In summary, the evidence for the benefit of VDS on outcomes related to the MetS in adults is inconclusive. Among the six indicators of MetS diagnosis, the results of this meta-analysis of RCTs showed that VDS intervention had no significant effect on WC, TG, HDL-C, and TC, but had improvement on BP and FPG. Further study is required before any accurate conclusions concerning the clinical importance of VDS in MetS can be reached. In the future, duration, the dosing regimen of supplementation and the target vitamin D levels remains an area for research.

## Data Availability

The original contributions presented in the study are included in the article/Supplementary Material, further inquiries can be directed to the corresponding author.
