# Supplementary material for: The impacts of vitamin D supplementation in adults with metabolic syndrome: A systematic review and meta-analysis of randomized controlled trials
Source: Front Pharmacol. 2022 Oct 5;13:1033026. doi: 10.3389/fphar.2022.1033026 (PMC9581173; doi:10.3389/fphar.2022.1033026)
Supplement: Supplementary file 1 [file Table1.DOCX]

**Supplement 1: Search strategy**

**Pubmed**

|  | Searches | Results |
| --- | --- | --- |
| #1 | Search: (((((((((((((((((((((((((vitamin d) OR (Cholecalciferol)) OR (Calciol)) OR ((3 beta,5Z,7E)-9,10-Secocholesta-5,7,10(19)-trien-3-ol)) OR (Vitamin D 3)) OR (Vitamin D3)) OR (Cholecalciferols)) OR (Hydroxycholecalciferols)) OR (Hydroxyvitamins D)) OR (Hydroxycholecalciferol)) OR (Ergocalciferols)) OR (Calciferols)) OR (Vitamin D 2)) OR (Vitamin D2)) OR (D2, Vitamin)) OR (Ergocalciferol)) OR (25-Hydroxyvitamin D 2)) OR (25 Hydroxyvitamin D 2)) OR (25-Hydroxyergocalciferol)) OR (25 Hydroxyergocalciferol)) OR (25-Hydroxyvitamin D2)) OR (25 Hydroxyvitamin D2)) OR (9,10-Secoergosta-5,7,10(19),22-tetraene-3 beta,25-diol)) OR (Ercalcidiol)) OR (25-Hydroxycalciferol)) OR (25 Hydroxycalciferol) Filters: Randomized Controlled Trial | **4913** |
| #2 | Search: ((((((((((((((((((((((Metabolic Syndrome) OR (Metabolic Syndromes)) OR (Syndrome, Metabolic)) OR (Syndromes, Metabolic)) OR (Metabolic Syndrome X)) OR (Insulin Resistance Syndrome X)) OR (Syndrome X, Metabolic)) OR (Syndrome X, Insulin Resistance)) OR (Metabolic X Syndrome)) OR (Syndrome, Metabolic X)) OR (X Syndrome, Metabolic)) OR (Dysmetabolic Syndrome X)) OR (Syndrome X, Dysmetabolic)) OR (Reaven Syndrome X)) OR (Syndrome X, Reaven)) OR (Metabolic Cardiovascular Syndrome)) OR (Cardiovascular Syndrome, Metabolic)) OR (Cardiovascular Syndromes, Metabolic)) OR (Syndrome, Metabolic Cardiovascular)) OR (Cardiometabolic Syndrome)) OR (Cardiometabolic Syndromes)) OR (Syndrome, Cardiometabolic)) OR (Syndromes, Cardiometabolic) Filters: Randomized Controlled Trial | **3446** |
| #3 | #1 AND #2 | **83** |
| #4 | Search: (((((((((((((((((((((((Metabolic Syndrome) OR (Metabolic Syndromes)) OR (Syndrome, Metabolic)) OR (Syndromes, Metabolic)) OR (Metabolic Syndrome X)) OR (Insulin Resistance Syndrome X)) OR (Syndrome X, Metabolic)) OR (Syndrome X, Insulin Resistance)) OR (Metabolic X Syndrome)) OR (Syndrome, Metabolic X)) OR (X Syndrome, Metabolic)) OR (Dysmetabolic Syndrome X)) OR (Syndrome X, Dysmetabolic)) OR (Reaven Syndrome X)) OR (Syndrome X, Reaven)) OR (Metabolic Cardiovascular Syndrome)) OR (Cardiovascular Syndrome, Metabolic)) OR (Cardiovascular Syndromes, Metabolic)) OR (Syndrome, Metabolic Cardiovascular)) OR (Cardiometabolic Syndrome)) OR (Cardiometabolic Syndromes)) OR (Syndrome, Cardiometabolic)) OR (Syndromes, Cardiometabolic) AND (randomizedcontrolledtrial[Filter])) AND ((((((((((((((((((((((((((vitamin d) OR (Cholecalciferol)) OR (Calciol)) OR ((3 beta,5Z,7E)-9,10-Secocholesta-5,7,10(19)-trien-3-ol)) OR (Vitamin D 3)) OR (Vitamin D3)) OR (Cholecalciferols)) OR (Hydroxycholecalciferols)) OR (Hydroxyvitamins D)) OR (Hydroxycholecalciferol)) OR (Ergocalciferols)) OR (Calciferols)) OR (Vitamin D 2)) OR (Vitamin D2)) OR (D2, Vitamin)) OR (Ergocalciferol)) OR (25-Hydroxyvitamin D 2)) OR (25 Hydroxyvitamin D 2)) OR (25-Hydroxyergocalciferol)) OR (25 Hydroxyergocalciferol)) OR (25-Hydroxyvitamin D2)) OR (25 Hydroxyvitamin D2)) OR (9,10-Secoergosta-5,7,10(19),22-tetraene-3 beta,25-diol)) OR (Ercalcidiol)) OR (25-Hydroxycalciferol)) OR (25 Hydroxycalciferol) AND (randomizedcontrolledtrial[Filter])) Filters: Randomized Controlled Trial, in the last 10 years | **73** |

**Web of Science**

|  | Searches | Results |
| --- | --- | --- |
| #1 | **(Metabolic Syndrome OR Syndrome, Metabolic OR Syndromes, Metabolic OR Metabolic Syndrome X OR Insulin Resistance Syndrome X OR Syndrome X, Metabolic OR Syndrome X, Insulin Resistance OR Metabolic X Syndrome OR Syndrome, Metabolic X OR X Syndrome, Metabolic OR Dysmetabolic Syndrome X OR Syndrome X, Dysmetabolic OR Reaven Syndrome X OR Syndrome X, Reaven OR Metabolic Cardiovascular Syndrome OR Cardiovascular Syndrome, Metabolic OR Cardiovascular Syndromes, Metabolic OR Syndrome, Metabolic Cardiovascular OR Cardiometabolic Syndrome OR Cardiometabolic Syndromes OR Syndrome, Cardiometabolic OR Syndromes, Cardiometabolic)** (Topic) | 334785 |
| #2 | **(((((TS= (vitamin d)) OR TS=(Cholecalciferol)) OR TS=(Hydroxycholecalciferols)) OR TS=(Ergocalciferols))) OR TS= (25-Hydroxyvitamin D 2)** | 218826 |
| #3 | #1 AND #2 | 6321 |
| #4 | **(PY= (2012-2022)) AND TS= (randomized controlled trial)** | 431655 |
| #5 | #3 AND #4 | **370** |

**The Cochrane Library**

ID Search Hits

#1 MeSH descriptor: [Vitamin D] explode all trees 6144

#2 (Cholecalciferol* or calciol or HYDROXYCHOLECALCIFEROL* or CALCIFEDIOL) 3938

#3 (calcidiol or DIHYDROXYCHOLECALCIFEROL* or CALCITRIOL) 2031

#4 (ergocalciferol* or Dihydrotachysterol or dihydroxyvitamin d or dihydroxyvitamins d or 1 alpha,25 dihydroxyvitamin d3): ti,ab,kw 2269

#5 (vitamin d): ti,ab,kw 15886

#6 (vitamin d2 or vitamin d 3): ti,ab,kw 9120

#7 #1 or #2 or #3 or #4 or #5 or #6 with Publication Year from 2012 to 2022, in Trials 11598

#8 (metabolic syndrome): ti,ab,kw 14874

#9 (syndrome near/1 metabolic or reaven* syndrome* or dysmetabolic near/1 syndrome* or X near/1 syndrome or Cardiovascular near/1 syndrome* or metabolic near/1 cardiovascular or metabolism near/1 syndrome* or insulin near/1 sensitivity or insulin near/1 resistance or hyperinsulin*): ti,ab,kw 25763

#10 #8 or #9 27200

#11 #7 AND #10 AND "randomized-controlled trials" **156**

**Embase**

(**'metabolic syndrome'**/exp OR **'metabolic syndrome'**) AND (**'vitamin d'**/exp OR **'vitamin d'**) AND (**'randomized controlled trial'**/exp OR **'randomized controlled trial'**) **311**

| **Supplement 2: GRADEing of the Meta-analysis** | | | | | | | | | | | |
| --- | --- | --- | --- | --- | --- | --- | --- | --- | --- | --- | --- |
| **Participants (studies) Follow-up** | **Risk of bias** | **Inconsistency** | **Indirectness** | **Imprecision** | **Publication bias** | **Overall certainty of evidence** | **Study event rates (%)** | | **Relative effect (95% CI)** | **Anticipated absolute effects** | |
|  |  |  |  |  |  |  | **With placebo** | **With VDS** |  | **Risk with placebo** | **Risk difference with VDS** |
| **25（OH）D** | | | | | | | | | | | |
| 879 (12 RCTs) | not serious | very serious^a^ | not serious | not serious | none | ⨁⨁◯◯ Low | 447 | 432 | - | The mean 25（OH）D was **0** | MD **17.41 higher** (14.09 higher to 20.73 higher) |
| **BMI** | | | | | | | | | | | |
| 645 (9 RCTs) | not serious | not serious | not serious | serious^b^ | none | ⨁⨁⨁◯ Moderate | 332 | 313 | - | The mean BMI was **0** | MD **0.27 higher** (0.06 lower to 0.59 higher) |
| **SBP** | | | | | | | | | | | |
| 357 (5 RCTs) | not serious | not serious | not serious | not serious | none | ⨁⨁⨁⨁ High | 188 | 169 | - | The mean SBP was **0** | MD **4.02 lower** (7.04 lower to 1.01 lower) |
| **DBP** | | | | | | | | | | | |
| 357 (5 RCTs) | not serious | not serious | not serious | not serious | none | ⨁⨁⨁⨁ High | 188 | 169 | - | The mean DBP was **0** | MD **3.11 lower** (4.91 lower to 1.3 lower) |
| **WC** | | | | | | | | | | | |
| 434 (5 RCTs) | not serious | not serious | not serious | very serious^c^ | none | ⨁⨁◯◯ Low | 226 | 208 | - | The mean WC was **0** | MD **0.29 lower** (2.03 lower to 1.46 higher) |
| **WHR** | | | | | | | | | | | |
| 144 (2 RCTs) | not serious | not serious | not serious | not serious | none | ⨁⨁⨁⨁ High | 72 | 72 | - | The mean WHR was **0** | MD **0.01 lower** (0.01 lower to 0 ) |
| **Body fat (%)** | | | | | | | | | | | |
| 245 (3 RCTs) | not serious | not serious | not serious | very serious^c^ | publication bias strongly suspected^d^ | ⨁◯◯◯ Very low | 123 | 122 | - | The mean body fat (%) was **0** | MD **0.1 higher** (0.09 lower to 0.3 higher) |
| **TC** | | | | | | | | | | | |
| 555 (9 RCTs) | not serious | not serious | not serious | serious^e^ | none | ⨁⨁⨁◯ Moderate | 287 | 268 | - | The mean TC was **0** | MD **4.94 lower** (11.4 lower to 1.51 higher) |
| **TG** | | | | | | | | | | | |
| 555 (9 RCTs) | not serious | not serious | not serious | serious^e^ | publication bias strongly suspected^d^ | ⨁⨁◯◯ Low | 287 | 268 | - | The mean TG was **0** | MD **7.87 lower** (16.82 lower to 1.09 higher) |
| **HDL-C** | | | | | | | | | | | |
| 555 (9 RCTs) | not serious | very serious^f^ | not serious | very serious^c^ | none | ⨁◯◯◯ Very low | 287 | 268 | - | The mean HDL-C was **0** | MD **0.61 lower** (3.77 lower to 2.56 higher) |
| **LDL-C** | | | | | | | | | | | |
| 555 (9 RCTs) | not serious | not serious | not serious | serious^e^ | none | ⨁⨁⨁◯ Moderate | 287 | 268 | - | The mean LDL-C was **0** | MD **1.85 lower** (7.16 lower to 3.46 higher) |
| **FPG** | | | | | | | | | | | |
| 757 (10 RCTs) | not serious | serious^g^ | not serious | not serious | publication bias strongly suspected^d^ | ⨁⨁◯◯ Low | 386 | 371 | - | The mean FPG was **0** | MD **3.78 lower** (6.52 lower to 1.03 lower) |
| **FI** | | | | | | | | | | | |
| 603 (9 RCTs) | not serious | very serious^h^ | not serious | serious^b^ | none | ⨁◯◯◯ Very low | 311 | 292 | - | The mean FI was **0** | MD **2.04 lower** (3.48 lower to 0.6 lower) |
| **HOMA-IR** | | | | | | | | | | | |
| 603 (9 RCTs) | not serious | not serious | not serious | very serious^c^ | none | ⨁⨁◯◯ Low | 311 | 292 | - | The mean HOMA-IR was **0** | MD **0.51 lower** (0.78 lower to 0.25 lower) |
| **QUICKI** | | | | | | | | | | | |
| 267 (4 RCTs) | not serious | serious^i^ | not serious | serious^b^ | none | ⨁⨁◯◯ Low | 135 | 132 | - | The mean QUICKI was **0** | MD **0.01 higher** (0 to 0.02 higher) |
| **HbA1C** | | | | | | | | | | | |
| 160 (2 RCTs) | not serious | not serious | not serious | very serious^c^ | none | ⨁⨁◯◯ Low | 88 | 72 | - | The mean hbA1C was **0** | MD **0.18 lower** (0.5 lower to 0.14 higher) |
| **MDA** | | | | | | | | | | | |
| 184 (2 RCTs) | serious^j^ | serious^i^ | not serious | not serious | none | ⨁⨁◯◯ Low^i^ | 100 | 84 | - | The mean MDA was **0** | MD **0.73 lower** (1.31 lower to 0.14 lower) |
| **hs-CRP** | | | | | | | | | | | |
| 212 (3 RCTs) | not serious | not serious | not serious | very serious^c^ | none | ⨁⨁◯◯ Low | 115 | 97 | - | The mean hs-CRP was **0** | MD **1.31 lower** (1.97 lower to 0.66 lower) |
| **PTH** | | | | | | | | | | | |
| 74 (74 RCTs) | not serious | not serious | not serious | not serious | none | ⨁⨁⨁⨁ High | 38 | 36 | - | The mean PTH was **0** | MD **7.35 lower** (19.18 lower to 4.48 higher) |
| **Serum Calcium** | | | | | | | | | | | |
| 200 (200 RCTs) | not serious | not serious | not serious | not serious | none | ⨁⨁⨁⨁ High | 98 | 102 | - | The mean serum Calcium was **0** | MD **0**  (0.13 lower to 0.13 higher) |

**CI:** confidence interval; **MD:** mean difference

#### Explanations

a. Downgraded by two level due to serious inconsistency. I2=95%

b. Downgraded by one level due to serious imprecision. Confidence interval includes both values suggesting benefit and no effect. Low number of participants.

c. Downgraded by two levels due to very serious imprecision. Confidence interval includes both values suggesting benefit and values suggesting harm. Low number of participants.

d. The funnel plot is asymmetric and the included RCTs were small samples

e. Downgraded by one level due to serious imprecision. Confidence interval includes both values suggesting benefit and harm. Low number of participants.

f. Downgraded by one level due to serious inconsistency. I2=72%

g. Downgraded by two level due to serious inconsistency. I2=49%

h. Downgraded by one level due to serious inconsistency. I2=52%

i. Downgraded by two level due to serious inconsistency. I2=43%

j. Downgraded by one level due to serious risk of bias. Unclear sequence generation and allocation concealment.
