# Supplementary material for: The impacts of vitamin D supplementation in adults with metabolic syndrome: A systematic review and meta-analysis of randomized controlled trials
Source: Front Pharmacol. 2022 Oct 5;13:1033026. doi: 10.3389/fphar.2022.1033026 (PMC9581173; doi:10.3389/fphar.2022.1033026)
Supplement: Supplementary file 2 [file DataSheet1.PDF]

Supplement 1: Search strategy

Pubmed

	Searches	Results
#1	Search: (((((((((((((((((((((((vitamin d) OR (Cholecalciferol)) OR (Calciol)) OR ((3 beta,5Z,7E)-9,10-Secocholesta-5,7,10(19)-trien-3-ol)) OR (Vitamin D 3)) OR (Vitamin D3)) OR (Cholecalciferols)) OR (Hydroxycholecalciferols)) OR (Hydroxyvitamins D)) OR (Hydroxycholecalciferol)) OR (Ergocalciferols)) OR (Calciferols)) OR (Vitamin D 2)) OR (Vitamin D2)) OR (D2, Vitamin)) OR (Ergocalciferol)) OR (25-Hydroxyvitamin D 2)) OR (25 Hydroxyvitamin D 2)) OR (25-Hydroxyergocalciferol)) OR (25 Hydroxyergocalciferol)) OR (25-Hydroxyvitamin D2)) OR (25 Hydroxyvitamin D2)) OR (9,10-Secoergosta-5,7,10(19),22-tetraene-3 beta,25-diol)) OR (Ercalcidiol)) OR (25-Hydroxycalciferol)) OR (25 Hydroxycalciferol) Filters: Randomized Controlled Trial	**4913**
#2	Search: ((((((((((((((((((((Metabolic Syndrome) OR (Metabolic Syndromes)) OR (Syndrome, Metabolic)) OR (Syndromes, Metabolic)) OR (Metabolic Syndrome X)) OR (Insulin Resistance Syndrome X)) OR (Syndrome X, Metabolic)) OR (Syndrome X, Insulin Resistance)) OR (Metabolic X Syndrome)) OR (Syndrome, Metabolic X)) OR (X Syndrome, Metabolic)) OR (Dysmetabolic Syndrome X)) OR (Syndrome X, Dysmetabolic)) OR (Reaven Syndrome X)) OR (Syndrome X, Reaven)) OR (Metabolic Cardiovascular Syndrome)) OR (Cardiovascular Syndrome, Metabolic)) OR (Cardiovascular Syndromes, Metabolic)) OR (Syndrome, Metabolic Cardiovascular)) OR (Cardiometabolic Syndrome)) OR (Cardiometabolic Syndromes)) OR (Syndrome, Cardiometabolic)) OR (Syndromes, Cardiometabolic) Filters: Randomized Controlled Trial	**3446**
#3	#1 AND #2	**83**
#4	Search: ((((((((((((((((((((Metabolic Syndrome) OR (Metabolic Syndromes)) OR (Syndrome, Metabolic)) OR (Syndromes, Metabolic)) OR (Metabolic Syndrome X)) OR (Insulin Resistance Syndrome X)) OR (Syndrome X, Metabolic)) OR (Syndrome X, Insulin Resistance)) OR (Metabolic X Syndrome)) OR (Syndrome, Metabolic X)) OR (X Syndrome, Metabolic)) OR (Dysmetabolic Syndrome X)) OR (Syndrome X, Dysmetabolic)) OR (Reaven Syndrome X)) OR (Syndrome X, Reaven)) OR (Metabolic Cardiovascular Syndrome)) OR (Cardiovascular Syndrome, Metabolic)) OR (Cardiovascular Syndromes, Metabolic)) OR (Syndrome, Metabolic Cardiovascular)) OR (Cardiometabolic Syndrome)) OR (Cardiometabolic Syndromes)) OR (Syndrome, Cardiometabolic)) OR (Syndromes, Cardiometabolic) AND (randomizedcontrolledtrial[Filter])) AND (((((((((((((((((((((((((vitamin d) OR (Cholecalciferol)) OR (Calciol)) OR ((3 beta,5Z,7E)-9,10-Secocholesta-5,7,10(19)-trien-3-ol)) OR (Vitamin D 3)) OR (Vitamin D3)) OR (Cholecalciferols)) OR (Hydroxycholecalciferols)) OR (Hydroxyvitamins D)) OR (Hydroxycholecalciferol)) OR (Ergocalciferols)) OR (Calciferols)) OR (Vitamin D 2)) OR (Vitamin D2)) OR (D2, Vitamin)) OR	**73**

	(Ergocalciferol)) OR (25-Hydroxyvitamin D 2)) OR (25 Hydroxyvitamin D 2)) OR (25-Hydroxyergocalciferol)) OR (25 Hydroxyergocalciferol)) OR (25-Hydroxyvitamin D2)) OR (25 Hydroxyvitamin D2)) OR (9,10-Secoergosta-5,7,10(19),22-tetraene-3 beta,25-diol)) OR (Ercalcidiol)) OR (25-Hydroxycalciferol)) OR (25 Hydroxycalciferol) AND (randomizedcontrolledtrial[Filter])) Filters: Randomized Controlled Trial, in the last 10 years	

Web of Science

	Searches	Results
#1	**(Metabolic Syndrome OR Syndrome, Metabolic OR Syndromes, Metabolic OR Metabolic Syndrome X OR Insulin Resistance Syndrome X OR Syndrome X, Metabolic OR Syndrome X, Insulin Resistance OR Metabolic X Syndrome OR Syndrome, Metabolic X OR X Syndrome, Metabolic OR Dysmetabolic Syndrome X OR Syndrome X, Dysmetabolic OR Reaven Syndrome X OR Syndrome X, Reaven OR Metabolic Cardiovascular Syndrome OR Cardiovascular Syndrome, Metabolic OR Cardiovascular Syndromes, Metabolic OR Syndrome, Metabolic Cardiovascular OR Cardiometabolic Syndrome OR Cardiometabolic Syndromes OR Syndrome, Cardiometabolic OR Syndromes, Cardiometabolic)** (Topic)	334785
#2	**(((((TS= (vitamin d)) OR TS=(Cholecalciferol)) OR TS=(Hydroxycholecalciferols)) OR TS=(Ergocalciferols))) OR TS= (25-Hydroxyvitamin D 2)**	218826
#3	#1 AND #2	6321
#4	**(PY= (2012-2022)) AND TS= (randomized controlled trial)**	431655
#5	#3 AND #4	**370**

The Cochrane Library

ID Search Hits

#1 MeSH descriptor: [Vitamin D] explode all trees 6144

#2 (Cholecalciferol* or calciol or HYDROXYCHOLECALCIFEROL* or CALCIFEDIOL) 3938

#3 (calcidiol or DIHYDROXYCHOLECALCIFEROL* or CALCITRIOL) 2031

#4 (ergocalciferol* or Dihydrotachysterol or dihydroxyvitamin d or dihydroxyvitamins d or 1 alpha,25 dihydroxyvitamin d3): ti,ab,kw 2269

#5 (vitamin d): ti,ab,kw 15886

#6 (vitamin d2 or vitamin d 3): ti,ab,kw 9120

#7 #1 or #2 or #3 or #4 or #5 or #6 with Publication Year from 2012 to 2022, in Trials 11598

#8 (metabolic syndrome): ti,ab,kw 14874

#9 (syndrome near/1 metabolic or reaven* syndrome* or dysmetabolic near/1 syndrome* or X near/1 syndrome or Cardiovascular near/1 syndrome* or metabolic near/1 cardiovascular or metabolism near/1 syndrome* or insulin near/1 sensitivity or insulin near/1 resistance or hyperinsulin*): ti,ab,kw 25763

#10 #8 or #9 27200

#11 #7 AND #10 AND "randomized-controlled trials" **156**

Embase

(**'metabolic syndrome'**/exp OR **'metabolic syndrome'**) AND (**'vitamin d'**/exp OR **'vitamin d'**) AND (**'randomized controlled trial'**/exp OR **'randomized controlled trial'**) **311**

Supplement 2: GRADEing of the Meta-analysis

Participants (studies) Follow-up	Risk of bias	Inconsistency	Indirectness	Imprecision	Publication bias	Overall certainty of evidence	Study event rates (%)		Relative effect (95% CI)	Anticipated absolute effects	
							With placebo	With VDS		Risk with placebo	Risk difference with VDS
**25 (OH) D**											
879 (12 RCTs)	not serious	very serious[a]	not serious	not serious	none	⊕⊕◯◯ Low	447	432	-	The mean 25 (OH) D was **0**	MD **17.41 higher** (14.09 higher to 20.73 higher)
**BMI**											
645 (9 RCTs)	not serious	not serious	not serious	serious[b]	none	⊕⊕⊕◯ Moderate	332	313	-	The mean BMI was **0**	MD **0.27 higher** (0.06 lower to 0.59 higher)
**SBP**											
357 (5 RCTs)	not serious	not serious	not serious	not serious	none	⊕⊕⊕⊕ High	188	169	-	The mean SBP was **0**	MD **4.02 lower** (7.04 lower to 1.01 lower)
**DBP**											
357 (5 RCTs)	not serious	not serious	not serious	not serious	none	⊕⊕⊕⊕ High	188	169	-	The mean DBP was **0**	MD **3.11 lower** (4.91 lower to 1.3 lower)
**WC**											
434 (5 RCTs)	not serious	not serious	not serious	very serious[c]	none	⊕⊕◯◯ Low	226	208	-	The mean WC was **0**	MD **0.29 lower** (2.03 lower to 1.46 higher)
**WHR**											
144 (2 RCTs)	not serious	not serious	not serious	not serious	none	⊕⊕⊕⊕ High	72	72	-	The mean WHR was **0**	MD **0.01 lower** (0.01 lower to 0)
**Body fat (%)**											
245 (3 RCTs)	not serious	not serious	not serious	very serious[c]	publication bias strongly suspected[d]	⊕◯◯◯ Very low	123	122	-	The mean body fat (%) was **0**	MD **0.1 higher** (0.09 lower to 0.3 higher)
**TC**											
555 (9 RCTs)	not serious	not serious	not serious	serious[e]	none	⊕⊕⊕◯ Moderate	287	268	-	The mean TC was **0**	MD **4.94 lower** (11.4 lower to 1.51 higher)

Supplement 2: GRADEing of the Meta-analysis

TG

555 (9 RCTs)	not serious	not serious	not serious	serious[e]	publication bias strongly suspected[d]	⊕⊕◯◯ Low	287	268	-	The mean TG was **0**	MD **7.87 lower** (16.82 lower to 1.09 higher)

HDL-C

555 (9 RCTs)	not serious	very serious[f]	not serious	very serious[c]	none	⊕◯◯◯ Very low	287	268	-	The mean HDL-C was **0**	MD **0.61 lower** (3.77 lower to 2.56 higher)

LDL-C

555 (9 RCTs)	not serious	not serious	not serious	serious[e]	none	⊕⊕⊕◯ Moderate	287	268	-	The mean LDL-C was **0**	MD **1.85 lower** (7.16 lower to 3.46 higher)

FPG

757 (10 RCTs)	not serious	serious[g]	not serious	not serious	publication bias strongly suspected[d]	⊕⊕◯◯ Low	386	371	-	The mean FPG was **0**	MD **3.78 lower** (6.52 lower to 1.03 lower)

FI

603 (9 RCTs)	not serious	very serious[h]	not serious	serious[b]	none	⊕◯◯◯ Very low	311	292	-	The mean FI was **0**	MD **2.04 lower** (3.48 lower to 0.6 lower)

HOMA-IR

603 (9 RCTs)	not serious	not serious	not serious	very serious[c]	none	⊕⊕◯◯ Low	311	292	-	The mean HOMA-IR was **0**	MD **0.51 lower** (0.78 lower to 0.25 lower)

QUICKI

267 (4 RCTs)	not serious	serious[i]	not serious	serious[b]	none	⊕⊕◯◯ Low	135	132	-	The mean QUICKI was **0**	MD **0.01 higher** (0 to 0.02 higher)

HbA1C

160 (2 RCTs)	not serious	not serious	not serious	very serious[c]	none	⊕⊕◯◯ Low	88	72	-	The mean hbA1C was **0**	MD **0.18 lower** (0.5 lower to 0.14 higher)

MDA

184 (2 RCTs)	serious[j]	serious[i]	not serious	not serious	none	⊕⊕◯◯ Low[i]	100	84	-	The mean MDA was **0**	MD **0.73 lower** (1.31 lower to 0.14 lower)

hs-CRP

Supplement 2: GRADEing of the Meta-analysis

212 (3 RCTs)	not serious	not serious	not serious	very serious[c]	none	⊕⊕◯◯ Low	115	97	-	The mean hs-CRP was **0**	MD **1.31 lower** (1.97 lower to 0.66 lower)

PTH

74 (74 RCTs)	not serious	not serious	not serious	not serious	none	⊕⊕⊕⊕ High	38	36	-	The mean PTH was **0**	MD **7.35 lower** (19.18 lower to 4.48 higher)

Serum Calcium

200 (200 RCTs)	not serious	not serious	not serious	not serious	none	⊕⊕⊕⊕ High	98	102	-	The mean serum Calcium was **0**	MD **0** (0.13 lower to 0.13 higher)

CI: confidence interval; **MD:** mean difference

Explanations

a. Downgraded by two level due to serious inconsistency. I2=95%
b. Downgraded by one level due to serious imprecision. Confidence interval includes both values suggesting benefit and no effect. Low number of participants.
c. Downgraded by two levels due to very serious imprecision. Confidence interval includes both values suggesting benefit and values suggesting harm. Low number of participants.
d. The funnel plot is asymmetric and the included RCTs were small samples
e. Downgraded by one level due to serious imprecision. Confidence interval includes both values suggesting benefit and harm. Low number of participants.
f. Downgraded by one level due to serious inconsistency. I2=72%
g. Downgraded by two level due to serious inconsistency. I2=49%
h. Downgraded by one level due to serious inconsistency. I2=52%
i. Downgraded by two level due to serious inconsistency. I2=43%
j. Downgraded by one level due to serious risk of bias. Unclear sequence generation and allocation concealment.
